# Reduction of Nitroarenes into Aryl Amines and *N*-Aryl hydroxylamines via Activation of NaBH_4_ and Ammonia-Borane Complexes by Ag/TiO_2_ Catalyst

**DOI:** 10.3390/nano6030054

**Published:** 2016-03-22

**Authors:** Dimitrios Andreou, Domna Iordanidou, Ioannis Tamiolakis, Gerasimos S. Armatas, Ioannis N. Lykakis

**Affiliations:** 1 Department of Chemistry, Aristotle University of Thessaloniki, University Campus, Thessaloniki 54124, Greece; dandreou@chem.auth.gr (D.A.); diordani@chem.auth.gr (D.I.); 2 Department of Materials Science and Technology, University of Crete, Vassilika Vouton, Heraklion 71003, Greece; gtam@materials.uoc.gr (I.T.); garmatas@materials.uoc.gr (G.S.A.)

**Keywords:** silver nanoparticles, nitroarenes, aryl amines, *N*-aryl hydroxylamines, titania, heterogeneous catalysis, selective reduction

## Abstract

In this study, we report the fabrication of mesoporous assemblies of silver and TiO_2_ nanoparticles (Ag/MTA) and demonstrate their catalytic efficiency for the selective reduction of nitroarenes. The Ag/TiO_2_ assemblies, which show large surface areas (119–128 m^2^·g^−1^) and narrow-sized mesopores (*ca.* 7.1–7.4 nm), perform as highly active catalysts for the reduction of nitroarenes, giving the corresponding aryl amines and *N*-aryl hydroxylamines with NaBH_4_ and ammonia-borane (NH_3_BH_3_), respectively, in moderate to high yields, even in large scale reactions (up to 5 mmol). Kinetic studies indicate that nitroarenes substituted with electron-withdrawing groups reduced faster than those with electron-donating groups. The measured positive ρ values from the formal Hammett-type kinetic analysis of *X*-substituted nitroarenes are consistent with the proposed mechanism that include the formation of possible [Ag]-H hybrid species, which are responsible for the reduction process. Because of the high observed chemo selectivities and the clean reaction processes, the present catalytic systems, *i.e.*, Ag/MTA-NaBH_4_ and Ag/MTA-NH_3_BH_3_, show promise for the efficient synthesis of aryl amines and *N*-aryl hydroxylamines at industrial levels.

## 1. Introduction

Noble metal nanoparticles are well known for their novel applications in the field of catalysis, biotechnology, bio-engineering and environmental remediation [1,2,3,4,5]. In recent years, the synthesis of noble metal nanoparticles as well as their applications, especially in catalysis, is of great interest for further study. Among them, silver nanoparticles (AgNPs) have attracted considerable attention because of their low cost, strong plasmonic properties and superior catalytic activity [6,7,8]. Several recent efforts to improve the physical properties of AgNPs have focused on synthesis of size- and shape-controlled nanoparticles, which were intended to enhance their catalytic and biological performance [6,7,8,9,10,11]. However, the practical use of individual AgNPs is often hampered by their severe aggregation during the catalytic reactions. In general, there are two main synthetic routes to overcome these limitations and to obtain small AgNPs with large exposed surface area. One is the surface modification of nanoparticles with organic molecules or surfactants in order to stabilize them against agglomeration and the other is the deposition of nanoparticles on a high-surface-area solid support such as metaloxides (SiO_2_, TiO_2_, ZrO_2_, *etc.*), modified carbons, graphene, or other porous materials. The latter seems to be the most preferred method to sustain the stability and catalytic activity of nanoparticles [6,7,8]. Ag loaded TiO_2_ (Ag/TiO_2_) nanocomposites have recently emerged as promising catalysts for use in organic synthesis, because of the combination between TiO_2_ electronic and optical properties, and Ag catalytic activities in chemical and biological areas. To this end, a variety of approaches have been used to prepare TiO_2_ supported Ag catalysts, for instance, by photo deposition, chemical deposition and conventional impregnation method [6,7,8,9,10,11,12]. In these studies, however, most of the Ag/TiO_2_ materials are composed of random aggregates of TiO_2_ and Ag nanoparticles [6,7,8]. Moreover, the TiO_2_ nanoparticles tend to agglomerate into bulk structures leading to a significant decrease in surface area and, therefore, in catalytic activity during repeated reactions.

Thus far, AgNPs have been successfully used as catalysts for several organic transformations, including C–C and C–N cross coupling reactions, cycloaddition and oxidative cyclization reactions, three-component reactions, oxidation of hydrosilanes to silanols, and reduction of imines to the corresponding amines [6,7,8,9,10,11]. In addition, there are several reports for the synthesis of AgNPs supported on different surfaces, which demonstrated their catalytic activity for the reduction of aromatic nitro compounds to the corresponding amines in the presence of sodium borohydride (NaBH_4_) as reducing agent [13,14,15,16,17,18,19,20,21,22]. To our knowledge, only few examples of AgNPs-based catalyst exhibiting high catalytic activity in nitroarenes reduction have been reported, although excess of NaBH_4_ or hast conditions (high temperature) are required [8,13,14,15,16]. In addition, most of these systems include reduction of nitrophenols by supported AgNPs in aqueous solution [8,17,18,19,20,21,22].

We recently reported the synthesis of mesoporous TiO_2_ nanoparticle assemblies (MTA) using a one-pot chemical route. The MTA was prepared through sol-gel polymerization reaction between TiCl_4_ and Ti(OPr)_4_ in the presence of polyoxoethylene cetyl ether (Brij-58) block copolymer template [23,24]. Herein, we report the synthesis of new mesoporous hetero structures consisting of titania and silver nanoparticles (Ag/MTA) and demonstrate the catalytic activity of these materials towards the selective reduction of nitroarenes. The obtained Ag/TiO_2_ assemblies show large internal surface area with narrow pore-size distribution and exhibit extraordinary activity for nitroarenes reduction. We present a systematic study of the reduction of several substituted nitroarenesto the corresponding anilines and *N*-aryl hydroxylamines, using NaBH_4_ and ammonia-borane (NH_3_BH_3_) complexes as the reducing agents. Furthermore, a detailed mechanistic route for the AgNP-catalyzed reduction of nitroarenes in the presence of NaBH_4_ or NH_3_BH_3_ is interpreted on the basis of kinetic analysis, as well as on the products identification by liquid chromatography-mass spectrometry (LC-MS) and nuclear magnetic resonance (NMR) spectroscopy.

## 2. Results and Discussion

### 2.1. Structural Properties of Ag/TiO_2_ Catalysts

In this work, we employed commercial available AgNO_3_ and AgOTf compounds, Degussa P (25) nanoparticles and mesoporous Ag-loaded TiO_2_ nanoparticle assemblies (Ag/MTA) as catalysts for the selective reduction of various nitro compounds. All commercial catalysts were used as received. Mesoporous Ag/MTA composites with AgNPs loading amounts of 2, 3, 4 and 7 wt % were prepared by photochemical deposition of AgNPs on the surface of nanoparticle-based mesoporous titania (MTA) [24] (see Supplementary Materials for details). Energy dispersive X-ray spectroscopy (EDS) analysis of the obtained products evidenced that the Ag loadings in the Ag/MTA composites are very close to those expected from the stoichiometry of the reactions (see Table S1, Supplementary Materials).

**Figure 1:**
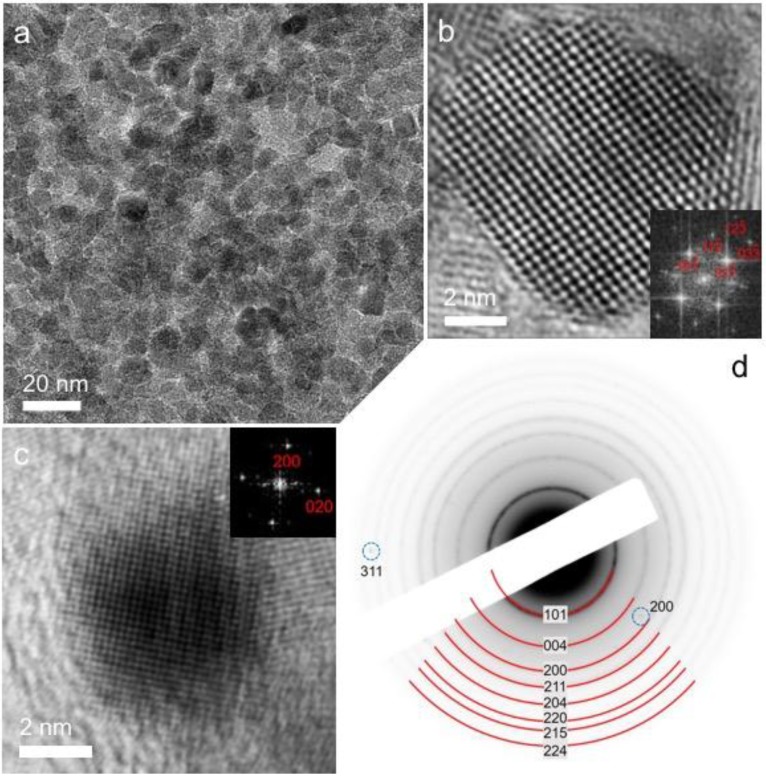
(**a**) Typical transmission electron microscopy (TEM) image; high resolution TEM (HRTEM) of a constituent (**b**) TiO_2_ and (**c**) Ag nanoparticle; and (**d**) selected-area electron diffraction (SAED) pattern of mesoporous 4% Ag/mesoporous TiO_2_ nanoparticle assemblies (MTA) catalyst. Insets of panels b and c: the corresponding fast Fourier transform (FFT) patterns indexed as the (111) and (100) zone axis diffraction of anatase TiO_2_ and face-centered cubic Ag, respectively.

The mesostructure and crystallinity of the Ag/MTA materials were characterized by X-ray diffraction (XRD), transmission electron microscopy (TEM) and nitrogen physisorption measurements. The powder XRD patterns (Figure S1, Supplementary Materials) indicated the well-defined crystal structure of Ag/MTA. They show a series of intense diffraction peaks in the 2θ = 20°–80° range, which can be assigned to the anatase structure of TiO_2_ (JCPDS #21-1272). In addition, the XRD patterns of Ag-loaded samples, especially those containing a high Ag loading (>3%), display weak diffractions at ~44.3° (002), ~64.5° (022) and ~77.4° (113), indicating the formation of metallic silver (JCPDS #04-0783). Figure 1a depicts a representative TEM image of the 4% Ag/MTA sample, which is the most active catalyst studied in this work. It reveals that this material consists of a porous network, which is composed of connected TiO_2_ and Ag nanoparticles. On the basis of this technique, the average size of TiO_2_ particles was estimated to be *ca.* 7–8 nm, while the diameter of Ag particles was found to be ~3–4 nm. Note that the average diameters of the TiO_2_ and Ag nanoparticles were estimated by counting more than 100 particles in several TEM images (Figure S2, Supplementary Materials). To investigate further the crystal structure of mesoporous network, high-resolution TEM (HRTEM) imaging and selected-area electron diffraction (SAED) were performed. The HRTEM images indicate the single-crystal structure of the constituting nanoparticles, showing well-resolved and continuous lattice fringes across the particles. The lattice fringes in Figure 1b,c can be readily assigned to the anatase TiO_2_ and face-centered cubic (fcc) Ag structure, respectively, in agreement with the XRD results. From the SAED pattern, it appeared that the crystalline structure of 4% Ag/MTA is a mixture of anatase TiO_2_ and cubic Ag. In agreement with XRD and HRTEM results, all the Debye–Scherrer diffraction rings can be reasonably assigned to the anatase phase of TiO_2_ (marker by red curves), while the additional diffraction spots could be indexed as (200) and (311) reflections of the cubic Ag (marker by blue dotted cycles) (Figure 1d).

The mesoporosity of the Ag/MTA materials was probed with N_2_ physisorption measurements at 77 K. The analysis showed that the Ag/MTA exhibit type-IV adsorption-desorption isotherms with H_2_-type hysteresis loop (Figure S3, Supplementary Materials), which correspond to mesoporous materials with narrow-sized pores. The Brunauer-Emmett-Teller (BET) surface area and total pore volume of the Ag/MTA were measured to be 119–128 m^2^·g^−1^ and 0.21–0.23 cm^3^·g^−1^, respectively, which are slightly lower than those of the parent TiO_2_ (MTA) sample (surface are ~149 m^2^·g^−1^ and pore volume ~0.27 cm^3^·g^−1^), probably due to the deposition of AgNPs on the TiO_2_ surface. The pore diameter in Ag/MTA samples was obtained by fitting the adsorption isotherms using the non-local density functional theory (NLDFT) model (assuming slit-like pores), and was found to be ~7.1–7.4 nm with narrow pore-size distribution. Furthermore, a weak peak at around 5.4–5.6 nm was observed, especially for the high-Ag-loaded samples (>3 wt %), which could be attributed to the pores filling with AgNPs. Table S1 in the Supplementary Materials summarizes all the textural properties of mesoporous MTA and Ag/MTA materials.

### 2.2. Evaluation of the Catalytic Activity

Initially, we proceeded to optimize the catalytic conditions by studying the reduction of 4-nitrotoluene (1). In Table 1, we show the yields of 4-toluidine (**1a**) obtained using different Ag-containing catalysts, reducing agents and solvents. Specifically, the catalyst (10 mg) was placed in a 5 mL glass reactor, followed by the addition of solvent (1 mL), nitroarene (0.1 mmol) and hydride compound, and the reaction was vigorous stirred for appropriate time. To our delight, we observed that the 4% Ag/MTA catalyst with 6 mol-excess of NaBH_4_ in ethanol affords quantitative conversion of **1** into 4-toluidine (**1a**) within 4 h (Table 1, entry 4).Remarkably, no by-products such as azoxy-, azo- or 1,2-diarylhydrazine were detected during the reaction progress by means of ^1^H-NMR. In comparison, lower reduction yields of **1a** were obtained with Ag/MTA catalysts containing 7 wt % or less than 3 wt % Ag, as shown in Table 1, entries 2, 3 and 5 (see also Supplementary Materials, Figure S4). Meanwhile, TiO_2_ alone, such as the MTA mesoporous and Degussa P25 nanoparticles (Table 1, entry 1) are largely inactive for the **1** reduction. Although, AgNO_3_ and AgOTf catalyzed the conversion of **1** in high yields and short reaction time (Table 1, entries 6 and 7), an equimolar amount of the sesaltsis necessary for the reaction completion. In contrast, silver wire does not catalyze any reduction process (Table 1, entry 8). The reaction was incomplete with lower amounts of NaBH_4_ (less than 2 mol-excess), while using 4 mol-excess of NaBH_4_ a prolonged reaction time of 24 h was required to obtain **1a** in >91% yield (results not shown). In addition, no reduction of **1** was observed under mild conditions, for example, using 1 bar of H_2_ at room temperature (Table 1, entry 9) or in the presence of transfer hydrogenation reagent such as the 1,1,3,3-tetramethyl disiloxane (TMDS) (Table 1, entry 10). In addition, with dimethylphenylsilane (DMPS), the conversion yield to the corresponding amine (**1a**) was only 11% (Table 1, entry 11). It is also noted that hydrazine hydrate (NH_2_NH_2_·H_2_O) can be activated under our catalytic conditions proceeding to quantitative reduction of **1**, but higher temperature and prolonged reaction time are required (Table 1, entry 12). In contrast, when ammonia-borane (NH_3_BH_3_) is used as reducing agent (2.5 mol-excess based on **1**) the corresponding *N*-aryl hydroxylamine (**1b**) was detected by ^1^H-NMR as the major product of **1** reduction, accompanying with small amount of amine **1a** (Table 1, entry 13). Control experiments showed no appreciable reduction of **1** in the absence of catalyst, indicating that the reduction process is catalytic in nature (result not shown). Finally, the reduction of **1** proceeded also efficiently in MeOH (Table 1, entry 14), while in other non-protic polar or apolar solvents no significant amount of **1a** was observed (Table 1, entries 15–19). Thus, 4% Ag/MTA catalyst in the presence of NaBH_4_ inethanol affords fast, quantitative, and clean reduction of **1** without the requirement of any chromatographic purification of the product **1a** (Table 1, entry 5).After completion of **1**, as evidenced by thin layer chromatography (TLC), the slurry was filtered through a short pad of silica to withhold the catalyst with the aid of ethanol (~5 mL). Then, the filtrate was evaporated under reduced pressure to give pure 4-toluidine **1a** (98% isolated yield) as a brown solid. It is worth noting that our catalytic conditions are significantly milder(room temperature and 6-mol excess of NaBH_4_) than those used in other studies, in which similar hydrogenation reactions were examined; however, either large excess of NaBH_4_ (~25–800 mol excess), or hast conditions (>100 °C) are required [13,14,15,16,17,18,19,20,21,22]. Based on this, our catalytic system is highly feasible for practical use.

### 2.3. Chemoselective Reduction of Nitroarenes into Aryl Amines and N-Aryl Hydroxylamines

**Scheme 1:**
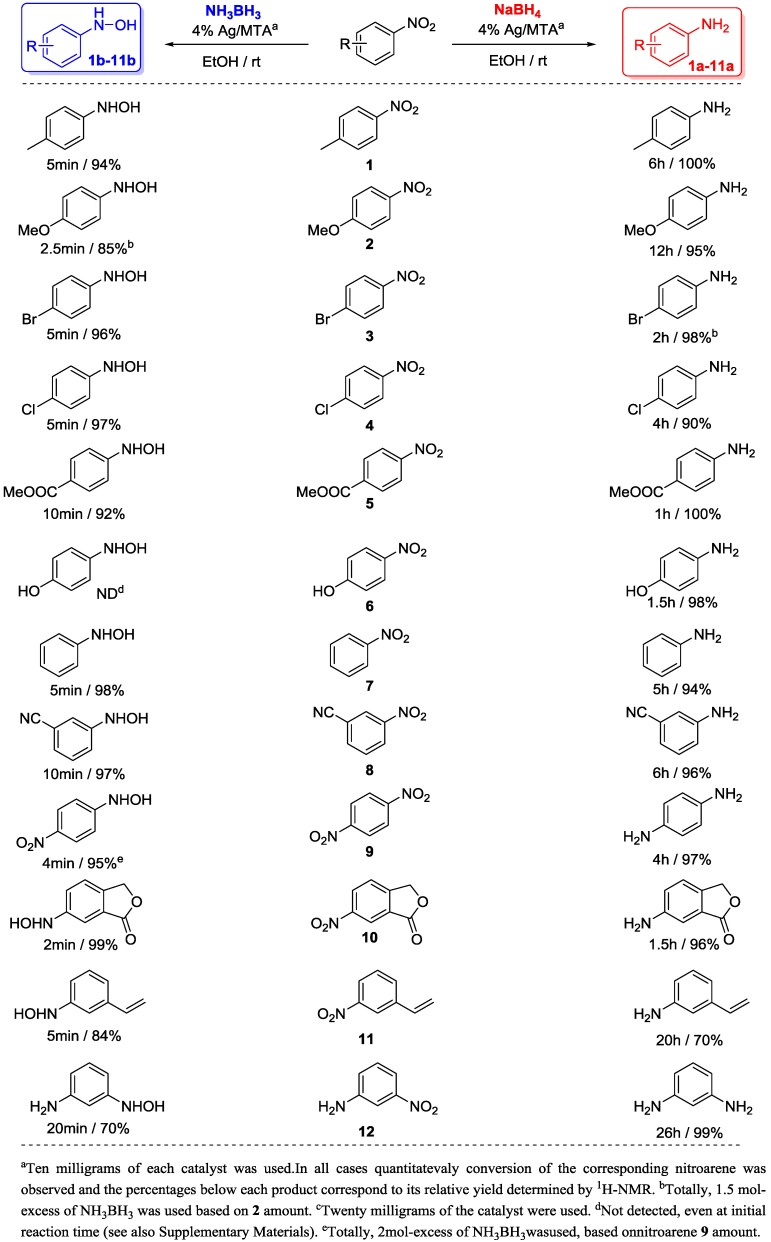
Chemoselective reduction of nitroarenes (**1**–**12**) into aryl amines (**1a**–**12a**) and *N*-aryl hydroxylamines (**1b**–**12b**) catalyzed by 4% Ag/MTA with NaBH_4_ and NH_3_BH_3_ complexes, respectively.

To study the limitation of this chemoselective reduction process, a series of nitroarenes (**1**–**12**) were examined under the above described conditions. As shown in Scheme 1, 4% Ag/MTA catalyst in the presence of NaBH_4_ (6 mol-excess based on nitroarene amount) produce the corresponding substituted anilines **1a**–**12a** in excellent yields (>90%) and selectivity (>98%). Moreover, bromo and chloro-substituted nitroarenes (**3** and **4**) were also reduced without undergoing any dehalogenation, while *p*-dinitrobenzene (**9**) was completely converted too diamine (**9a**) within 4 h. Similarly, carboxylate, and cyano functionalities in **5** and **8** nitroarenes remained intact under the examined conditions, indicating highly chemoselective reduction. Consistent with the above results, the reduction of 6-nitro-isobenzofuran-1(3*H*)-one (**10**) gave also the corresponding amine without further transformation of the lacton ring. Notably, 3-nitrostyrene **11** was reduced to the corresponding saturated amine **11a** in 70% relative yield, accompanying with a 3-ethyl-nitrobenzene yield of 30% (see Supplementary Materials). This result suggests that the present catalytic system is also active for the preferred hydrogenation of the C=C double bond relative to the nitro group of **11**.

Nevertheless, when the NH_3_BH_3_ (1.5–2.5 mol-excess based on nitroarene amount) is used as the reducing agent, the corresponding *N*-aryl hydroxylamines (**1b**–**12b**) are formed under the above described conditions (Scheme 1); indeed, in high relative yields (>84%) and within short reaction times (2–10 min). ^1^H-NMR analysis of the crude mixtures showed only a small amount of the corresponding substituted anilines (**1a**–**10a**) (2%–10%)as byproducts (see Supplementary Materials). As shown in Scheme 1, the reduction of *p*-nitrophenol (**6**) produces the corresponding amine **6a** as the only product. In this case, *N*-aryl hydroxylamine **6b** was not detected by NMR at initial reaction time (see Supplementary Materials, Figure S5). Reduction of **11** leads to the *N*-aryl hydroxylamine **11b** accompynaning with small amount of the initial nitroarene, however, under the described conditions **12** gave the **12b** in 70% relative yield with a significant amount of the corresponding amine **12a** (30%), (see Supplementary Materials). It should be noted that the reaction products were kept at room temperature and no further chromatographic purification was performed to avoid decomposition or transformation of the *N*-aryl hydroxylamines into the corresponding nitrosoarenes, anilines and azoxy- or azo-arenes. In the literature, several synthetic routes towards *N*-aryl hydroxylamines formation have been reported; however, certain limitations related to the applicability of these reactions (including scale-up synthesis) have been imposed. Since the first procedure using Zn/NH_4_Cl [25] and the enzymatic nitroreductase system [26], the common synthetic routes associated with the title transformation have focused on the direct hydrogenation (with H_2_) of nitroarenes using precious metal nanoparticles such as Pd, Pt, Ru, Re and Rh as catalysts [27,28,29,30,31,32,33]. In addition, catalytic transfer hydrogenation reactions of nitroarenes to *N*-aryl hydroxylamines were also realized using Pt/hydrazine [34], Zn in CO/H_2_O [35] and Sb/NaBH_4_ [36] systems, while only recently Au/TiO_2_ nanoparticles have been employed for the selective nitroalkanes reduction to *N*-alkyl hydroxylamines [37]. To our knowledge, since now, heterogeneous Ag-catalyzed hydrogenation of nitroarenes to the corresponding *N*-aryl hydroxylamines is an unknown transformation. These results, accompanying with the observation that *N*-aryl hydroxylamines are formed in high relative yields and through afast and pure reaction process, suggest that the present catalytic system Ag/MTA-NH_3_BH_3_ can be applicable to various hydrogenation reactions, including fine synthesis of *N*-aryl hydroxylamines.

### 2.4. Kinetic Studies

The reusability of the 4% Ag/MTA was examined by conducting repeat catalytic experiments. The 4% Ag/MTA catalyst can be easily separated from the reaction mixture by simply filtration and it can be reused for the next catalytic run. As shown in Supplementary Materials, Figure S6, the catalyst can be used at least three times without significant loss of its catalytic activity and selectivity. In order to assess the feasibility of the present catalytic system, the 4% Ag/MTA catalyst was further tested for large-scale production of aryl amines and *N*-aryl hydroxylamines from nitroarenes. For this reason, 5 mmol of nitroarene **1** were reduced in the presence of 4% Ag/MTA (0.8 mol %) with 8 mol-excess of NaBH_4_ in 15 mL EtOH. After completion of the reaction (within ~20 h based on TLC analysis), the mixture was filtered upon silica gel, washed with ethanol and purified by column chromatography to afford the corresponding 4-toluedine **1a** in 91% isolated yield. As a comparison, under the same reaction conditions but with 3 mol-excess of NH_3_BH_3_, the corresponding *N*-aryl hydroxyamine **1b** was obtained at 93% relative yield in 30 min, according to the ^1^H-NMR analysis of the crude product (results not shown). These results correspond to a turn over number (TON) of about 125 and a turnover frequency (TOF) of 250 h^−1^.

**Figure 2:**
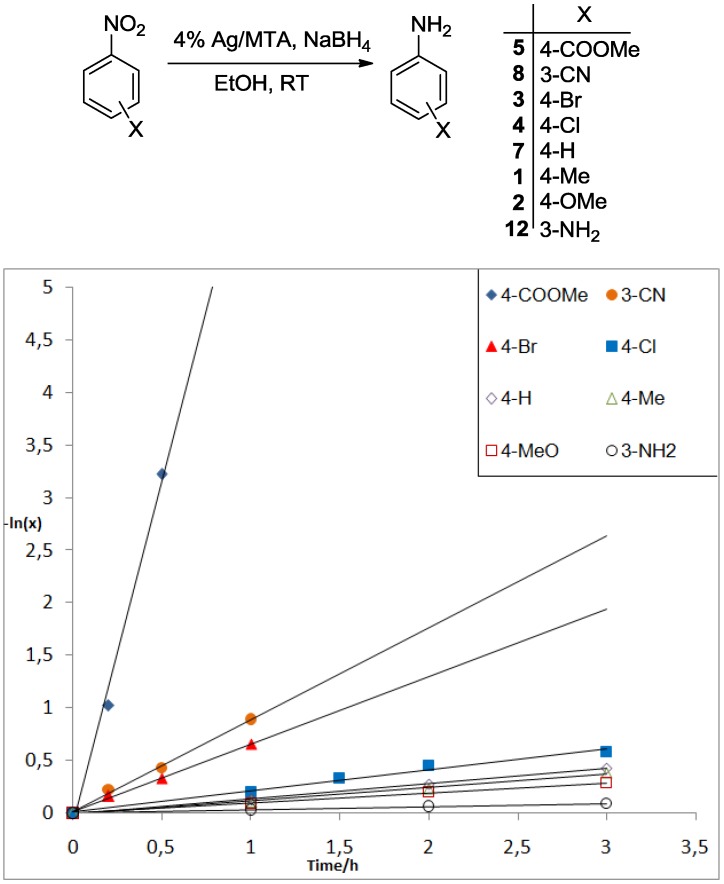
Kinetic analysis of the 4% Ag/MTA-catalyzed reduction of various *X*-substituted nitroarenes (X = 3-NH_2_ (**12**), 4-MeO (**2**), 4-Me (**1**), 4-Br (**3**), 4-H (**7**), 4-Cl (**4**), 3-CN (**8**) and 4-COOMe (**5**)) with NaBH_4_.

To propose a plausible mechanistic pathway for the present Ag-catalyzed reduction of nitroarenes, we performed a Hammett-type kinetic study for the reduction of a diverse set of *para*- and meta-*X*-substituted-nitroarenes (**1**, **2**, **3**, **4**, **5**, **7**, **8** and **12**). The kinetic studies were carried out as follow: 0.2 mmol of the nitroarene, 20 mg of 4% Ag/MTA and 0.6 mmol of NaBH_4_ were added in ethanol (1 mL). A 100 μL aliquot of the mixture was taken at appropriate time and the mixture was filtrated through a short pad of silica (to withhold the catalyst) and washed with ethanol (~1 mL). Then, the filtrate was evaporated under reduced pressure and the consumptions of the corresponding *X*-substituted nitroarene were determined by integrating the appropriate proton signals in ^1^H-NMR spectra. Each reaction was repeated at least three times and the average values are depicted in Figure 2 and in the Supplementary Materials, Figures S7 and S8. Considering that the concentration of the possible formed silver-hydride species remains constant at initial times and assuming a pseudo-first order dependence of the reaction rate on the nitroarene concentration, Equation (1) can be applied.

(1) ln(*x*)= −*kt*

where, *k* is the rate constant and *x* is the consumption of the *X*-substituted nitroarene at reaction time *t*.

According to Equation (1), a plot of the ln(*x*) *versus* time gives a linear curve, the slope of which is equal to the rate constant *k*. The results indicated that the kinetic activity of **1**, **2**, **3**, **4**, **5**, **7**, **8** and **12** nitroarenes is remarkably affected by the nature of the *X*-substituent group, in which the reduction proceeds faster as the electron-withdrawing ability of the substituent group increases. For example, the reduction of **3** (4-Br), **4** (4-Cl), **5** (4-COOMe) and **8** (3-CN) proceeds in a faster rate than that with the nitrobenzene **7** (X = H) (Figure 2), as indicated by the relative rate constant ratios *k*_COOMe_/*k*_H_ = 25, *k*_CN_/*k*_H_ = 6.0, *k*_Br_/*k*_H_ = 4.5 and *k*_Cl_/*k*_H_ = 1.4, respectively (see also Supplementary Materials, Figures S7 and S8). However, nitroarenes containing electron-donating group, such as **12** (3-NH_2_), **2** (4-MeO) and **1** (4-Me), were reduced with slower reaction rate (Figure 2); the relative rate constant rations were *k*_NH2_/*k*_H_ = 0.2, *k*_MeO_/*k*_H_ = 0.7 and *k*_Me_/*k*_H_ = 0.9, respectively. In addition to these results, a Hammett-type correlation in the competition of *X*-substituted nitroarenes (**12**, **2**, **1**, **4**, **3**, **8** and **5**) *versus* nitrobenzene (7) gave positive slopes, *i.e.*, ρ ≈ 0.8, *R*^2^ = 0.890 (using σ^+^ values) and ρ ≈ 0.9, *R*^2^ = 0.826 (using σ values) (Supplementary Materials, Figure S9). The small ρ values for these correlations indicate that the reaction mechanism involves either radical intermediates or a transition state with a small charge separation [38]. However, the measured positive ρ values, are consistent with a proposed mechanism that include a negative charge (or hydride transfer) in the transition state, which is stabilized by electron-withdrawing substituents [38,39].

According to the above results, we propose a general mechanistic pathway for the AgNPs catalyzed reduction of nitroarenes. First, a B-H bond cleavage occurs to gives [Ag]-H active species. Such hybrid species are responsible for the rapid reduction of nitroarenes into the corresponding *N*-aryl hydroxylamines. This assumption is consistent with the kinetics studies showing that nitroarenes bearing electron-withdrawing substituents reduced faster than those with electron donating groups. Finally, *N*-aryl hydroxylamines are further reduced into the corresponding aryl amines with NaBH_4_; however, this step becomes slower in the presence of NH_3_BH_3_. To shed light on the above hypothesis, the catalytic reductions of electron-donating **2** and electron-withdrawing **5** and **9** *p*-*X*-substituted nitroarenes were also separately conducted in CD_3_OD using 4% Ag/MTA. Each reduction process was monitored directly by ^1^H-NMR spectroscopy, at initial reaction times. As shown in Figures S10–S12 of the Supplementary Materials, during the reduction process, the only intermediate products were the corresponding *N*-aryl hydroxylamines **2b**, **5b** and **9b**.

## 3. Experimental Section

### 3.1. Materials

Brij 58 surfactant (HO(CH_2_CH_2_O)_20_C_16_H_33_, Mn~1124), titanium tetrachloride (99.9%), AgNO_3_ (>99%), AgOTf, absolute ethanol (99.8%), NaBH_4_, NH_3_BH_3_, TMDS and DMPS were purchased from Sigma-Aldrich (Darmstadt, Germany). Titanium(IV) isopropoxide (>98%) was purchased from Merck (Darmstadt, Germany). TiO_2_ nanoparticles (P25) were purchased from Degussa AG (Dusseldorf, Germany).The aromatic nitro compounds used as substrates were of high purity and commercially available from Aldrich (Darmstadt, Germany).

### 3.2. Synthesis of Ag/MTA Catalysts

The mesoporous TiO_2_ nanoparticle assemblies (MTA) were prepared according to the method reported previous [24]. Ag-loaded TiO_2_ (Ag/MTA) catalysts with different loading of AgNPs were obtained by photocatalytic reduction method. Typically, 0.2 g of MTA were dispersed into 10 mL of a CH_3_CN/H_2_O/ethanol (10:1:1 *v*/*v*) solution containing appropriate amounts of AgNO_3_. The suspension was then illuminated with a 5 mW ultraviolet lamp (λ = 365 nm) for 2 h under continuous stirring. The product was then collected by filtration, washed with ethanol, and dried at 60 °C for 12 h. A series of mesoporous Ag/MTA catalysts with different Ag loadings, *i.e.*, *x* = 2, 3, 4 and 7 wt %, was prepared using 6.4, 9.6, 13.2 and 23.8 mg of AgNO_3_, respectively.

### 3.3. Physical Characterization

The X-ray diffraction (XRD) patterns were collected using a Panalytical X’Pert Pro MPD X-ray diffractometer (45 kV and 40 mA, Lelyweg, the Netherlands) with a Cu Kα radiation (λ = 1.5406 Å). Elemental microprobe analysis was performed on a JEOL Model JSM-6390LV scanning electron microscopy (SEM, Tokyo, Japan) system equipped with an Oxford INCA PentaFET-x3 energy-dispersive X-ray spectroscopy (EDS) detector (Oxfordshire, UK). Data acquisition was performed several times using an accelerating voltage of 20 kV and 60 s accumulation time. Transmission electron microscopy (TEM) experiments were carried out with a JEOL model JEM-2100 electron microscope (LaB_6_ filament) operating at 200 kV. The sample was dispersed in ethanol by sonication, and the dispersion was then dropped onto a Cu grid covered with carbon film. Nitrogen adsorption-desorption isotherms were measured at liquid N_2_ temperature (77 K) on a NOVA 3200e volumetric analyzer (Quantachrome, Boynton Beach, FL, USA). Before analysis, samples were degassed overnight at 150 °C under vacuum (<10^−5^ Torr) to remove moisture. The specific surface areas were calculated using the Brumauer-Emmett-Teller (BET) method [40] on the adsorption data in the 0.06–0.25 relative pressure (*P*/*P*_o_) range. The total pore volumes were derived from the adsorbed volume at *P*/*P*_o_ = 0.99 and the pore size distributions were obtained by the nonlocal density functional theory (NLDFT) method [41] based on the adsorption data.

### 3.4. Catalytic Reactions

Supported silver catalyst Ag/MTA (10 mg) was placed in a 5 mL glass reactor, followed by the addition of ethanol (1 mL), nitro compound (0.1 mmol) and NaBH_4_ (0.6 mmol) or NH_3_BH_3_ (0.25 mmol), and the reaction mixture was stirred at room temperature for a selected time. The reaction was monitored by thin layer chromatography (TLC), and after completion, the slurry was filtered under pressure through a short pad of silica to withhold the catalyst with the aid of ethanol or methanol (~5 mL). After solvent evaporation the corresponding products were formed in pure forms. Product analysis was conducted by ^1^H-NMR and ^13^C-NMR spectroscopy (Bruker AM 300, Bruker Biospin GMBH, Rheinstetten, Germany and Agilent AM 500, Agilent Technologies, Santa Clara, CA, USA). Identification of the products was realized by comparing the NMR spectra with those of the commercially available pure substances. LC-MS 2010 EV Instrument (Shimadzu, Tokyo, Japan) under Electrospray Ionization (ESI) conditions was used for the determination of the mass spectra.

Reusability testing of the catalyst was conducted in the case of the 4% Ag/MTA sample through the reduction of 4-nitrotoluene (1). A 2 mL mixture of the feeding solution (0.2 mmol of **1**, 1.2 mmol of NaBH_4_ and 20 mg of catalyst (4 mol % Ag)) was placed into a vial. Each catalytic reaction was stopped after 6 h and the catalyst was collected by filtration, washed with ethanol and dried in an oven at 100 °C for 12 h. Then, the recovered catalyst was used for the next catalytic run without any additional treatment.

## 4. Conclusions

In conclusion, we have shown that mesoporous titania supported silver nanoparticles (Ag/MTA) can effectively catalyze the chemoselective reduction of nitroarenes into the corresponding aryl amines and *N*-aryl hydroxylamines, employing NaBH_4_ and NH_3_BH_3_ as reducing agents, respectively. Product analysis and kinetic studies indicated that aryl amine formation proceeds through a reduction pathway involving the initial formation of silver-hydride species; although additional mechanistic studies are required in this direction. In both catalytic processes, the corresponding *N*-aryl hydroxylamines were observed either as intermediates (with NaBH_4_) or as the major products (with NH_3_BH_3_). Based on the observed high chemoselectivities and the fast and clean reaction processes, both catalytic systems, Ag/MTA-NaBH_4_ and Ag/MTA-NH_3_BH_3_, can be applicable to various hydrogenation reactions, including fine synthesis of amines and *N*-aryl hydroxylamines, respectively.

## Figures and Tables

**Table 1:** Evaluation of various Ag-containing catalysts, reducing agents and solvents in the catalytic reduction of 4-nitrotoluene (**1**) into 4-toluidine (**1a**). 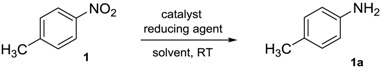

Entry	Catalyst ^1^	Solvent	Reducing agent ^2^	Time/Yield ^3^
1	MTA or P25	EtOH	NaBH_4_	24h/0%
2	2% Ag/MTA	EtOH	NaBH_4_	4h/10% ^4^
3	3% Ag/MTA	EtOH	NaBH_4_	4h/51% ^4^
**4**	**4% Ag/MTA**	**EtOH**	**NaBH_4_**	**4h/>99%**
5	7% Ag/MTA	EtOH	NaBH_4_	4h/30% ^4^
6	AgOTf	EtOH	NaBH_4_	0.5h/>99% ^5^
7	AgNO_3_	EtOH	NaBH_4_	0.5h/>99%^5^
8	Ag (wire)	EtOH	NaBH_4_	24h/0%
9	4% Ag/MTA	EtOH	H_2_ (1 atm)	24h/0%
10	4% Ag/MTA	EtOH	TMDS	24h/0%
11	4% Ag/MTA	EtOH	DMPS	4h/11%
12	4% Ag/MTA	EtOH	NH_2_NH_2_·H_2_O	24%/99% ^6^
**13**	**4% Ag/MTA**	**EtOH**	**NH_3_BH_3_**	**0.2h/6% (94%) ^7^**
14	4% Ag/MTA	MeOH	NaBH_4_	4h/99%
15	4% Ag/MTA	Toluene	NaBH_4_	4h/0%
16	4% Ag/MTA	THF	NaBH_4_	4h/0%
17	4% Ag/MTA	CH_3_CN	NaBH_4_	4h/0%
18	4% Ag/MTA	DCM	NaBH_4_	4h/0%
19	4% Ag/MTA	Acetone	NaBH_4_	4h/0%

^1^ Ten milligrams of each catalyst was used. ^2^ sixe mol-excess of NaBH_4_ were used, while 1,1,3,3-tetramethyl disiloxane (TMDS), dimethylphenylsilane (DMPS) and hydrazine were added in 2.5, 5 and 12 fold-excess of mmols, respectively, based on **1**. ^3^ Relative yield of **1a** determined by ^1^H-NMR. ^4^ The conversions of **1** were in the range of 75%–100%, while the azoxy-, azo- and hydrazo-arenes were formed as major products. ^5^ Using lower amount of the AgNO_3_ and AgOTf (20% mol and 50% mol, based on **1**) the conversions of **1** were in the range of 10%–79% after 2h; however, the corresponding hydrazo- and azo-arenes were formed as the major products accompynaning with small amount of the amine **1**. ^6^ Twelve mol-excess of hydrazine were used for reaction completion at 90 °C. ^7^ The corresponding *N*-aryl hydroxylamine **1b** was formed as the major product accompanying with small amount of the amine **1a**, within 10 min.

## Supplementary Materials

They are available online at http://www.mdpi.com/2079-4991/6/3/54/s1.
