# Supplementary material for: Reduction of Nitroarenes into Aryl Amines and N-Aryl hydroxylamines via Activation of NaBH4 and Ammonia-Borane Complexes by Ag/TiO2 Catalyst
Source: Nanomaterials (Basel). 2016 Mar 22;6(3):54. doi: 10.3390/nano6030054 (PMC5302523; doi:10.3390/nano6030054)
Supplement: Supplementary file 1 [file nanomaterials-06-00054-s001.pdf]

Supplementary Materials: Reduction of Nitroarenes into Aryl Amines and *N*-Aryl hydroxylamines via Activation of $NaBH_4$ and Ammonia-Borane Complexes by Ag/TiO_2 Catalyst

Dimitrios Andreou, Domna Iordanidou, Ioannis Tamiolakis, Gerasimos S. Armatas and Ioannis N. Lykakis

Table S1. Textural properties of mesoporous mesoporous TiO_2 nanoparticle assemblies (MTA) and Ag/MTA catalysts.

Sample	Ag loading [a] (wt %)	Surface area (m²/g)	Pore volume (cm³/g)	Pore size (nm)
MTA		149	0.27	7.1
2% Ag/MTA	2.1	126	0.23	7.1
3% Ag/MTA	2.9	128	0.23	5.6, 7.3
4% Ag/MTA	4.0	125	0.22	5.5, 7.4
7% Ag/MTA	6.8	119	0.21	5.4, 7.4

[a] Weight percent of Ag loading according to the Energy dispersive X-ray spectroscopy (EDS) data.

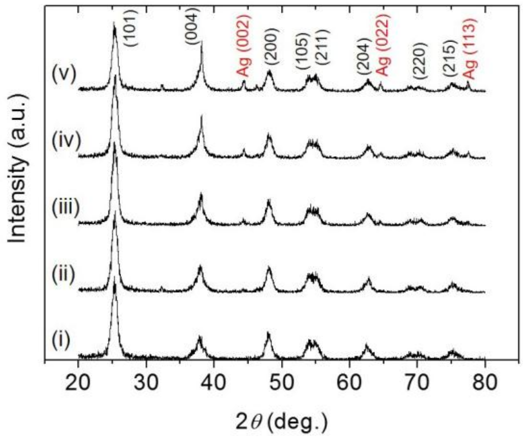

Figure S1. X-ray diffraction (XRD) patters of mesoporous samples: (**i**) MTA; (**ii**) 2% Ag/MTA; (**iii**) 3% Ag/MTA; (**iv**) 4% Ag/MTA; and (**v**) 7% Ag/MTA.

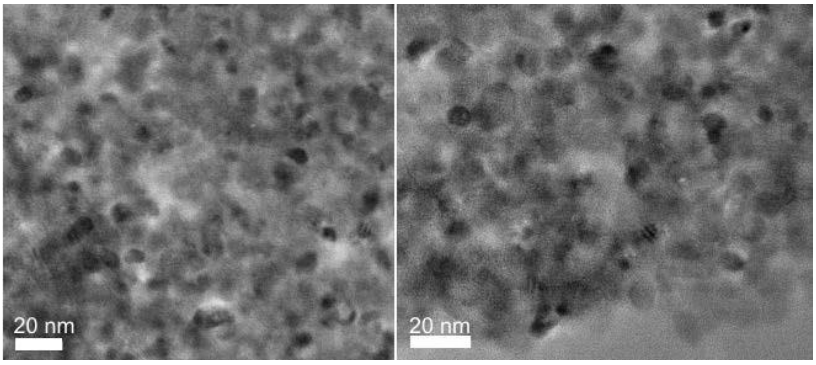

Figure S2. Typical transmission electron microscopy (TEM) images of mesoporous 4% Ag/MTA sample, showing that individual AgNPs (appeared as dark spots as evidenced by high resolution TEM (HRTEM) and fast Fourier transformation (FFT) analysis (see Figure 1c in the text)) are uniformly dispersed on the surface of TiO_2.

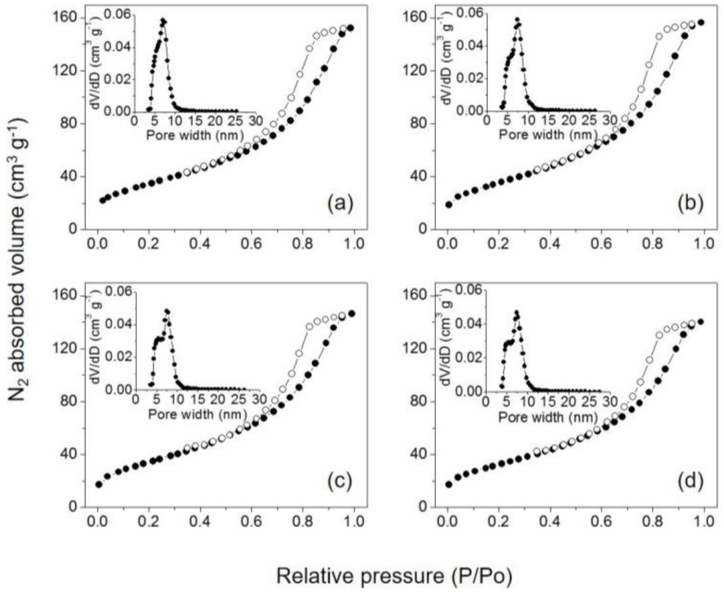

Figure S3. N_2 adsorption-desorption isotherms at 77 K and the corresponding non-local density functional theory (NLDFT) pore size distributions (insets) for the mesoporous: (**a**) 2% Ag/MTA; (**b**) 3% Ag/MTA; (**c**) 4% Ag/MTA; and (**d**) 7% Ag/MTA materials.

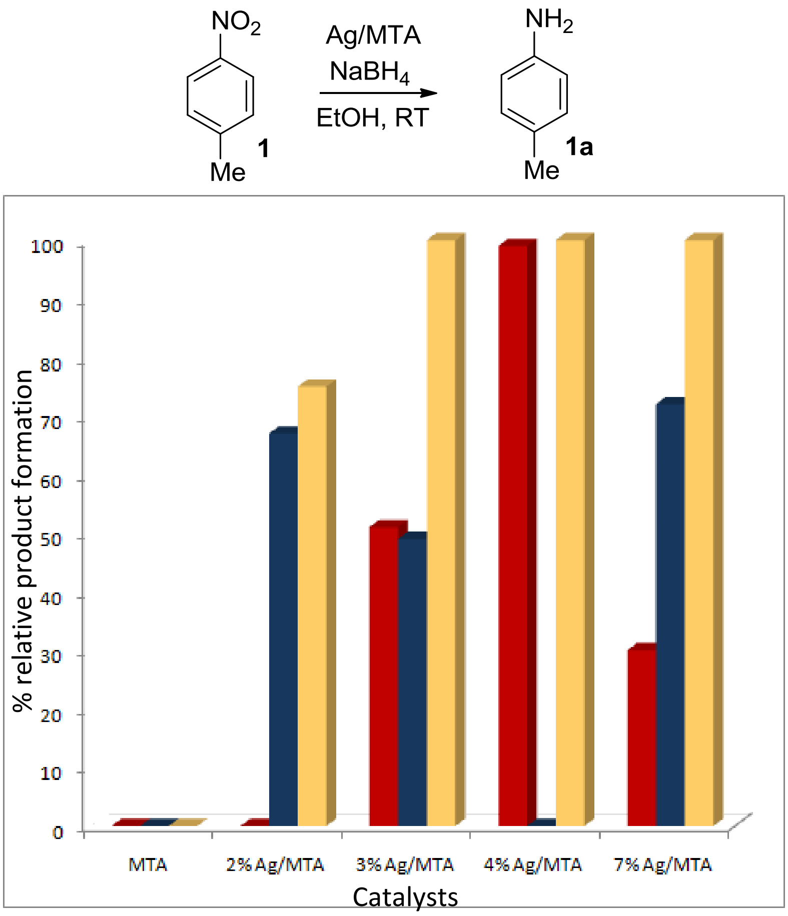

Figure S4. Catalysts evaluation based on the relative conversions of (**1**) to amine or to the condensation products azo, azoxy and hydrazo arenes, after 4 h. First (red) columns correspond to the relative yield of amine (**1a**); second (blue) columns correspond to the summary of the relative yields of the dimeric products; and third (yellow) columns correspond to the reaction conversion based on the consumption of **1**.

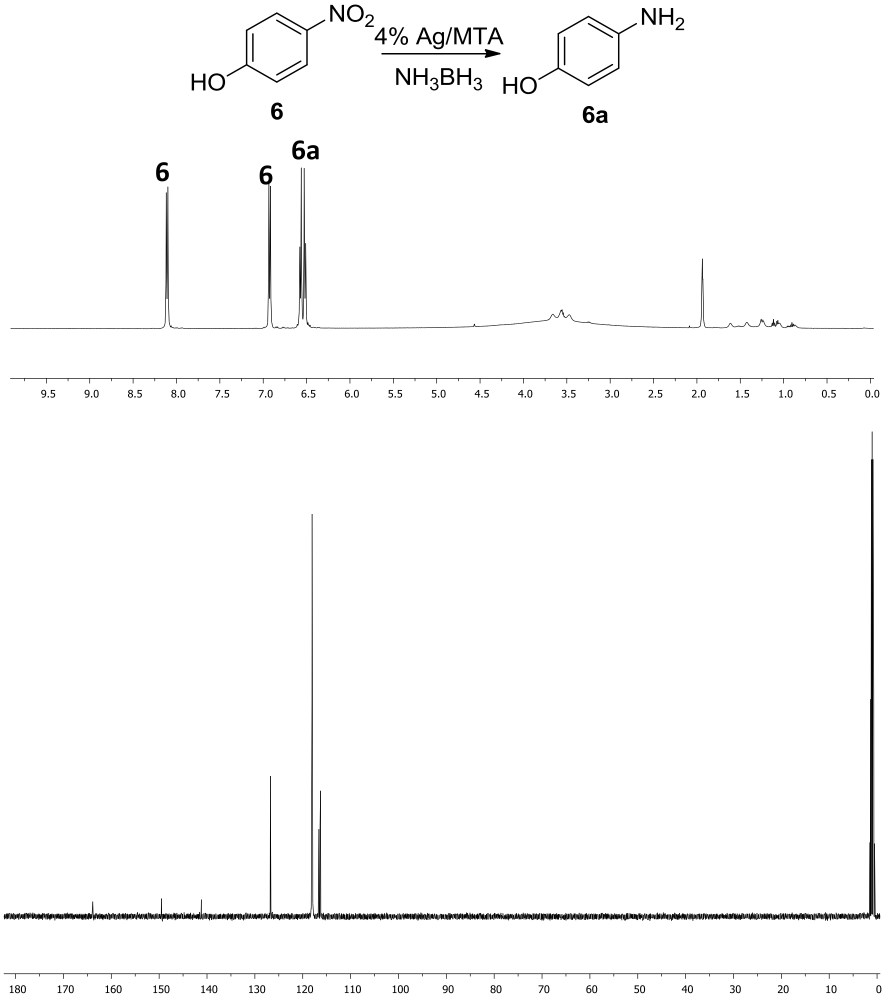

Figure S5. ^{1}H-NMR and ^{13}C-NMR of the crude mixture of the reduction of 6 in the presence of NH_3BH_3 catalyzed by 4% Ag/MTA.

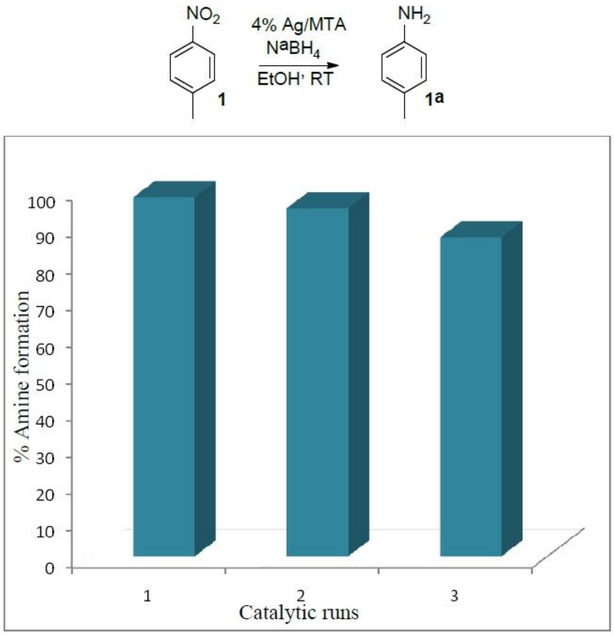

Figure S6. Recycling study of the mesoporous 4% Ag/MTA catalyst (Experimental conditions: 0.2 mmol of p-nitrotoluene, 20 mg of catalyst, 1.2 mmol of $NaBH_4$, 2 mL of ethanol, room temperature, *t* = 6 h).

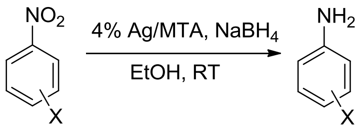

	X
**5**	4-COOMe
**8**	3-CN
**3**	4-Br
**4**	4-Cl
**7**	4-H
**1**	4-Me
**2**	4-OMe
**12**	3-NH_2

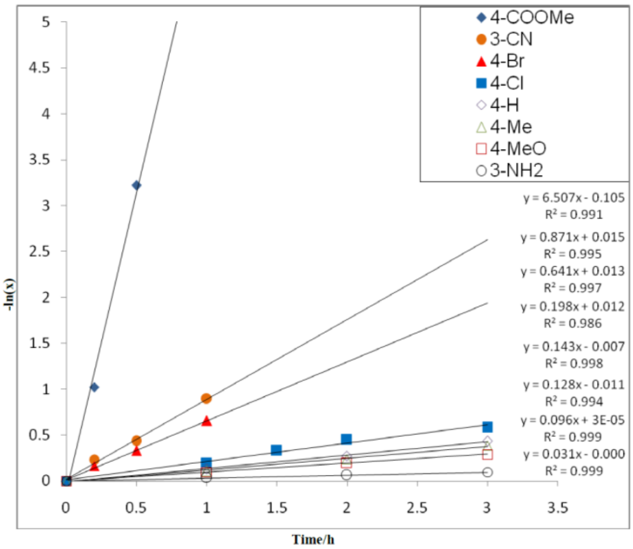

Figure S7. Kinetic plots for the nitroarenes **1**, **2**, **3**, **4**, **5**, **7**, **8** and **12** consumptions catalyzed by 4% Ag/MTA using $NaBH_4$ (4 mol-excess) as reducing agent.

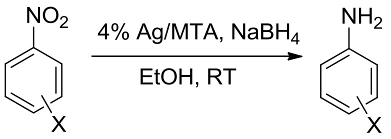

	X
**5**	4-COOMe
**8**	3-CN
**3**	4-Br
**4**	4-Cl
**7**	4-H
**1**	4-Me
**2**	4-OMe
**12**	3-NH_2

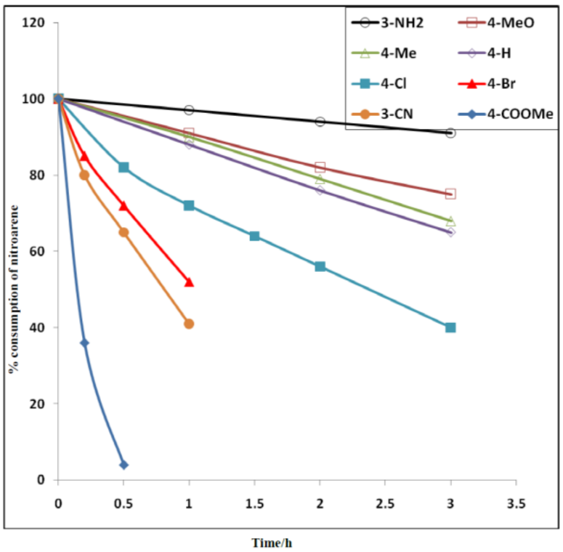

Figure S8. Profile of the nitroarenes **1**, **2**, **3**, **4**, **5**, **7**, **8** and **12** consumptions catalyzed by 4% Ag/MTA using $NaBH_4$ (4 mol-excess) as reducing agent.

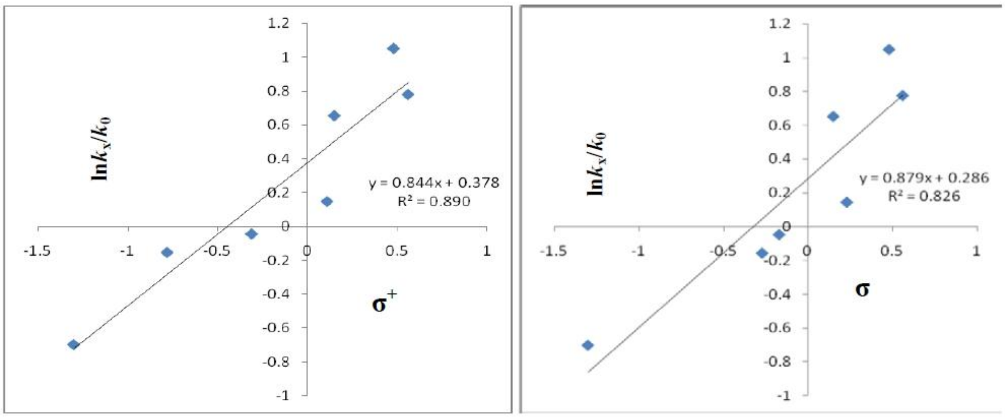

Figure S9. Hammett-type kinetic plots for the nitroarenes **1**, **2**, **3**, **4**, **5**, **8** and **12** reductions catalyzed by 4% Ag/MTA using $NaBH_4$ as reducing agent. The values for σ^+ and σ were taken from the textbook [1].

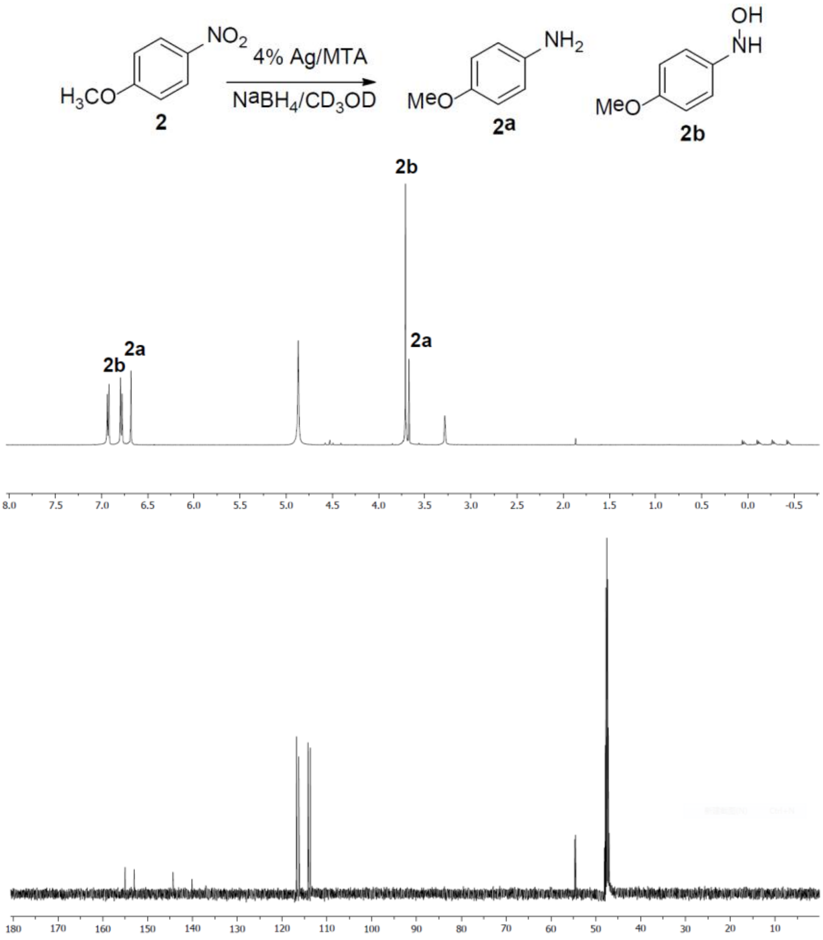

Figure S10. ^{1}H-NMR and ^{13}C-NMR of the crude mixture of the reduction of **2** in the presence of NaBH4 in CD_3OD, catalyzed by 4% Ag/MTA, at initial reaction time (<1 h).

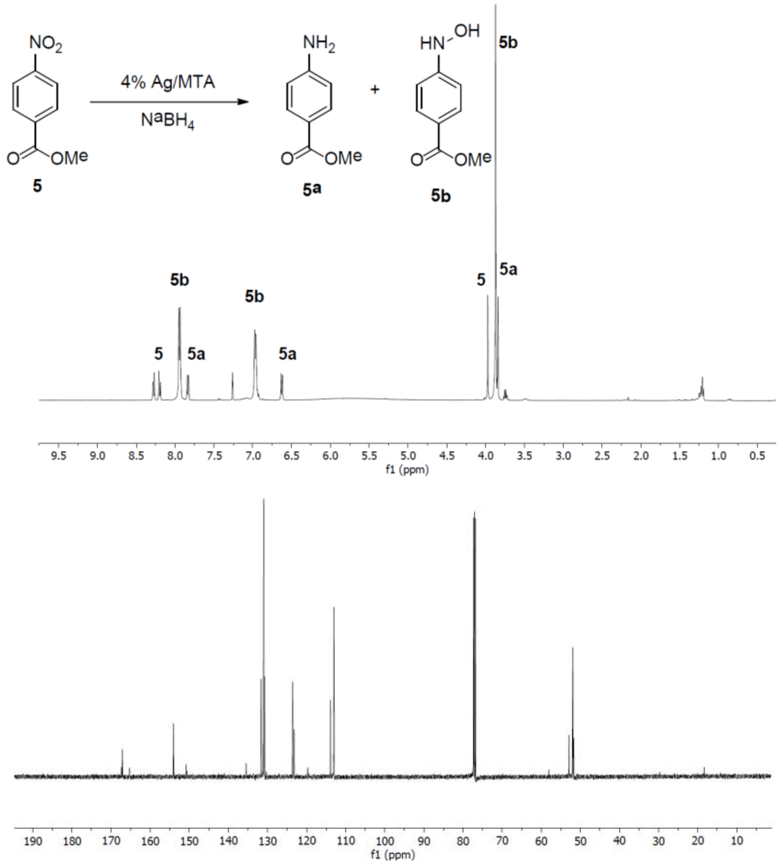

Figure S11. ^{1}H-NMR and ^{13}C-NMR of the crude mixture of the reduction of **5** in the presence of $NaBH_4$, catalyzed by 4% Ag/MTA, at initial reaction time (<0.5 h).

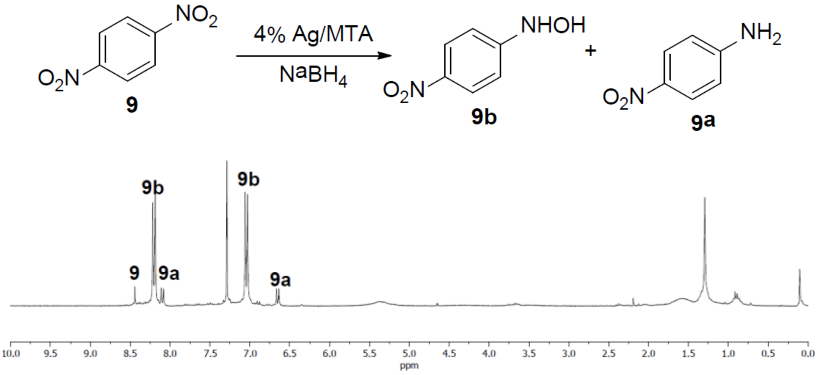

Figure S12. ^{1}H-NMR of the crude mixture of the reduction of **9** in the presence of $NaBH_4$, catalyzed by 4% Ag/MTA, at initial reaction time (<0.5 h).

^{1}H-NMR and ^{13}C-NMR Data

Toluidine [2,3]

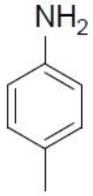

1a

^{1}H-NMR (300 MHz, $CDCl_3$): 6.91 (d, 2H, *J* = 8.5 Hz), 6.56 (d, 2H, *J* = 8.5 Hz), 3.49 (br, 2H, $-NH_2$), 2.19

(s, 3H); ^{13}C-NMR (75 MHz, $CDCl_3$): 143.6, 129.5, 127.6, 115.1, 20.2.

4-Methoxyaniline [2]

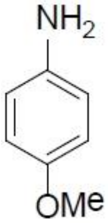

2a

^{1}H-NMR (300 MHz, $CDCl_3$): 6.75 (d, 2H, J = 9 Hz), 6.65 (d, 2H, J = 9 Hz), 3.74 (s, 3H), 3.48 (br, 2H, -NH_2); ^{13}C-NMR (75 MHz, $CDCl_3$): 152.8, 139.9, 116.4, 114.7, 55.7.

4-Bromoaniline [3]

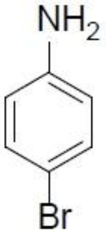

3a

^{1}H-NMR (300 MHz, $CDCl_3$): 7.22 (d, 2H, J = 8.5 Hz), 6.53 (d, 2H, J = 8.5 Hz), 3.65 (br, 2H, -NH_2); ^{13}C-NMR (75 MHz, $CDCl_3$): 145.4, 132.0, 116.7, 110.2.

4-Chloroaniline [3]

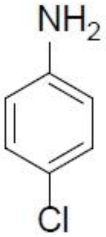

4a

^{1}H-NMR (300 MHz, $CDCl_3$): 7.04 (d, 2H, J = 8.5 Hz), 6.64 (d, 2H, J = 8.5 Hz), 3.62 (br, 2H, -NH_2); ^{13}C-NMR (75 MHz, $CDCl_3$): 144.8, 128.9, 123.0, 116.0.

Methyl 4-aminobenzoate [4]

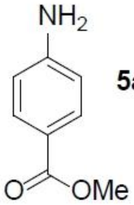

5a

^{1}H-NMR (300 MHz, $CDCl_3$): 7.83 (d, 2H, J = 8.5 Hz), 6.62 (d, 2H, J = 8.5 Hz), 4.09 (br, 2H, -NH_2), 3.84 (s, 3H); ^{13}C-NMR (75 MHz, $CDCl_3$): 167.2, 150.8, 131.5, 119.8, 113.7, 51.5.

4-Aminophenol [3]

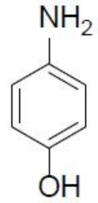

6a

^{1}H-NMR (300 MHz, CD_3OD): 6.56 (d, 2H, *J* =9 Hz), 6.51 (d, 2H, *J* = 9 Hz), 3.40 (br, 2H, $-NH_2$); ^{13}C-NMR (75 MHz, CD_3OD): 149.7, 141.7, 116.8, 116.7.

Aniline [2]

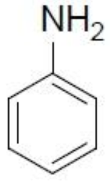

7a

^{1}H-NMR: (300 MHz, $CDCl_3$): 7.13 (t, 2H, *J* = 8.5 Hz), 6.73 (t, 1H, *J* = 8.5 Hz), 6.65 (d, 2H, *J* = 8.5 Hz), 3.42 (br, 2H, $-NH_2$); ^{13}C-NMR: (75 MHz, $CDCl_3$): 146.2, 129.0, 118.3, 114.9.

3-Aminobenzonitrile [5]

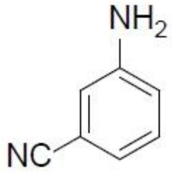

8a

^{1}H-NMR (300 MHz, $CDCl_3$): 7.18 (t, 1H, *J* = 7.5 Hz), 6.96 (d, 1H, *J* = 7.5 Hz), 6.89 (s, 1H), 6.85 (d, 1H, *J* = 7.5 Hz), 3.86 (br, 2H, $-NH_2$); ^{13}C-NMR (75 MHz, CDCl3): 146.9, 129.9, 121.8, 119.2, 119.1, 117.5, 112.9.

4-Aminoaniline [2]

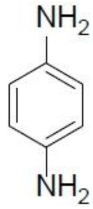

9a

^{1}H-NMR (300 MHz, $CDCl_3$): 6.56 (s, 4H), 3.19 (br, 4H, $-NH_2$); ^{13}C-NMR (75 MHz, $CDCl_3$): 138.6, 116.7.

6-aminoisobenzofuran-1(3*H*)-one [6]

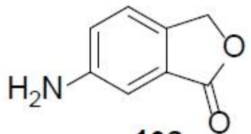

10a

^{1}H-NMR (300 MHz, $CDCl_3$): 7.21 (d, 1H, *J* = 8.0 Hz), 7.13 (d, 1H, *J* = 2.0 Hz), 6.95 (dd, 1H, J_1 = 8.0 Hz, J_2 = 2.0 Hz), 5.20 (s, 2H), 3.92 (s, 2H); ^{13}C-NMR (125 MHz, $CDCl_3$): 171.4, 147.5, 136.4, 127.0, 122.7, 121.6, 109.8, 69.6.

3-Ethylaniline [2,3]

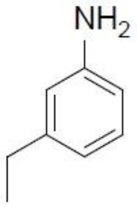

11a

^{1}H-NMR (300 MHz, $CDCl_3$): 7.07 (t, 1H, *J* = 7.5 Hz), 6.61 (d, 1H, *J* = 7.5 Hz), 6.54 (s, 1H), 6.51 (d, 1H, *J* = 7.5 Hz), 3.56 (s, 2H), 2.56 (q, 2H, *J* = 7 Hz), 1.21 (t, 3H, *J* = 7 Hz).

1-Ethyl-3-nitrobenzene

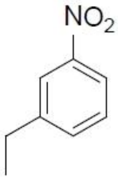

11c

^{1}H-NMR (300 MHz, $CDCl_3$): 7.76-7.72 (m, 1H), 7.43 (t, 1H, *J* =7.7 Hz), 7.31 (d, 2H, *J* = 7.7 Hz), 2,77 (q, 2H, *J* = 7.5 Hz). 1.32 (t, 3H, *J* = 7.5 Hz).

3-amino aniline [3,4]

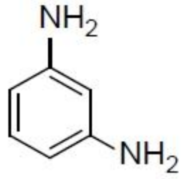

12a

^{1}H-NMR (500 MHz, $CDCl_3$): 6.94 (t, 1H, *J* = 7.9 Hz), 6.12 (dd, 2H, J_1 = 7.9 Hz, J_2 = 2.0 Hz), 6.04 (t, 1H, *J* = 2.0 Hz), 3.56 (br, 4H, $-NH_2$); ^{13}C-NMR (125 MHz, $CDCl_3$): 147.5, 130.2, 106.0, 101.9.

N-(p-tolyl)hydroxylamine [7,8]

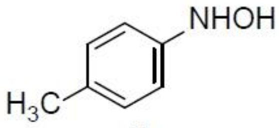

1b

^{1}H-NMR (300 MHz, $CDCl_3$): 7.09 (d, 2H, *J* = 8.4 Hz), 6.92 (d, 2H, *J* = 8.4 Hz), 2.30. ^{13}C-NMR (75 MHz, $CDCl_3$): 147.3, 132.0, 129.5, 115.2, 20.6.

N-(4-methoxyphenyl)hydroxylamine [7,8]

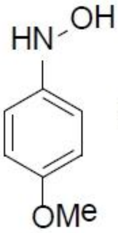

2b

^{1}H-NMR (300 MHz, CD_3OD): 6.94 (d, 2H, *J* = 9 Hz), 6.80 (d, 2H, *J* = 9 Hz), 3.72 (s, 3H); ^{13}C-NMR (125 MHz, CD_3OD): 156.5, 145.8, 118.3, 115.7, 56.1; MS *m*/*z* (ESI) calcd for $C_7H_9NO_2$ $(M-H)^+$ 138.06,

found 137.85.

N-(4−bromophenyl)hydroxylamine [7]

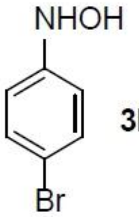

3b

^{1}H-NMR (300 MHz, $CDCl_3$): 7.38 (d, 2H, *J* = 8.7 Hz), 6.88 (d, 2H, *J* = 8.7 Hz), 5.51 (br, 1H). ^{13}C-NMR (75 MHz, $CDCl_3$): 148.9, 131.9, 116.2, 114.5.

N-(4-chlorophenyl)hydroxylamine [7]

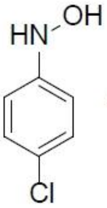

4b

^{1}H-NMR (300 MHz, $CDCl_3$): 7.24 (d, 2H, *J* = 8.8 Hz), 6.93(d, 2H, *J* = 8.8 Hz). ^{13}C-NMR (75 MHz, $CDCl_3$): 148.3, 128.9, 127.2, 115.9. ^{1}H-NMR (500 MHz, CD_3OD): 7.17 (d, 2H, *J* = 8.5 Hz), 6.93 (d, 2H, *J* = 8.5 Hz); ^{13}C-NMR (125 MHz, CD_3OD): 151.8, 129.5, 126.2, 116.2. MS *m*/*z* (ESI) calcd for C_6H_6NOCl $(M\text{-}H)^+$ 142.56, found 142.41.

Methyl 4-(hydroxyamino)benzoate [7]

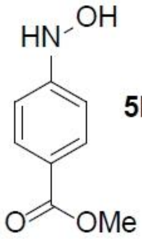

5b

^{1}H-NMR (300 MHz, $CDCl_3$): 7.96 (d, 2H, *J* = 8.4 Hz), 6.98 (d, 2H, *J* = 8.4 Hz), 3.88. ^{13}C-NMR (75 MHz, $CDCl_3$): 167.0, 154.0, 131.0, 123.5, 113.0, 51.8. ^{1}H-NMR (300 MHz, CD_3OD): 7.85 (d, 2H, *J* = 8.5 Hz), 6.93 (d, 2H, *J* = 8.5 Hz), 3.87 (s, 3H). MS *m*/*z* (ESI) calcd for $C_8H_9NO_3$ $(M\text{-}H)^+$ 166.05, found 165.90.

N-phenylhydroxylamine [7]

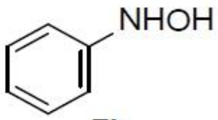

7b

^{1}H-NMR (300 MHz, $CDCl_3$): 7.31–7.28 (m, 2H), 7.02–6.97 (m, 3H). ^{13}C-NMR (75 MHz, $CDCl_3$): 149.7, 128.9, 122.4, 114.8.

3−(Hydroxyamino)benzonitrile [7,8]

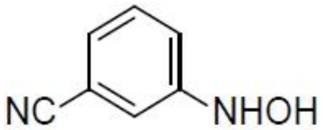

8b

^{1}H-NMR (500 MHz, $CDCl_3$): 7.34 (t, 1H, *J* = 8.0 Hz), 7.29 (s, 1H), 7.23 (d, 1H, *J* = 8.0 Hz), 7.15 (d, 1H, *J* = 8.0 Hz). ^{13}C-NMR (125 MHz, $CDCl_3$): 150.4, 129.6, 125.5, 118.9, 118.3, 117.1, 112.7. MS *m*/*z* (ESI) calcd for $C_7H_6N_2O$ $(M\text{-}H)^+$ 133.04, found 132.95.

N-(4-nitrophenyl)hydroxylamine [7]

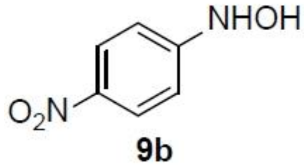

9b

^{1}H-NMR (300 MHz, Acetone-d_6): 8.11 (d, 2H, *J* = 9.2 Hz), 7.03 (d, 2H, *J* = 9.2 Hz). ^{13}C-NMR (75 MHz, Acetone-d_6): 157.9, 140.7, 126.0, 112.0.

6-(hydroxyamino)isobenzofuran-1(3H)-one [7,8]

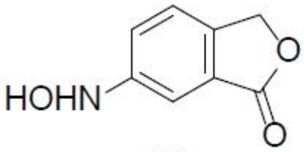

10b

^{1}H-NMR (500 MHz, Acetone-d_6): 8.15 (br, 1H), 7.46 (d, 1H, *J* = 8.3 Hz), 7.39 (s, 1H, *J* = 2.0 Hz), 7.31 (dd, 1H, J_1 = 8.3 Hz, J_2 = 1.7 Hz), 5.27 (s, 2H). ^{13}C-NMR (125 MHz, Acetone-d_6): 171.7, 148.6, 139.7, 127.2, 123.5, 120.9, 108.7, 70.3.

N-(3−Vinylphenyl)hydroxylamine [7]

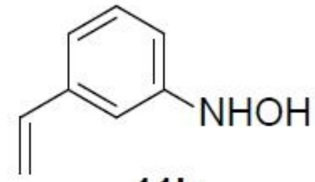

11b

^{1}H-NMR (500 MHz, CD_3OD): 7.16 (t, 1H, *J* = 7.5 Hz), 7.05 (s, 1H), 6.93 (d, 1H, *J* = 7.5 Hz), 6.84 (d, 1H, *J* = 7.5 Hz), 6.67 (dd, 1H, J_1 = 18 Hz, J_2 = 12 Hz), 5.69 (d, 1H, *J* = 18 Hz), 5.15 (d, 1H, *J* = 12 Hz); ^{13}C-NMR (125 MHz, CD_3OD): 153.0, 139.8, 138.6, 130.2, 120.2, 116.4, 114.2, 113.3; MS *m*/*z* (ESI) calcd for C_8H_9NO $(M\text{-}H)^+$ 134.06, found 133.95.

3-N-hydroxyl-aniline [7,8]

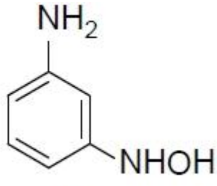

12b

^{1}H-NMR (500 MHz, $CDCl_3$): 7.04 (t, 1H, *J* = 8.0 Hz), 6.38 (s, 1H), 6.36 (d, 1H, *J* = 8.0 Hz), 6.31 (d, *J* = 8.0 Hz); ^{13}C-NMR (125 MHz, $CDCl_3$): 151.0, 147.2, 129.8, 109.2, 104.9, 101.4

^{1}H-NMR and ^{13}C-NMR Spectra

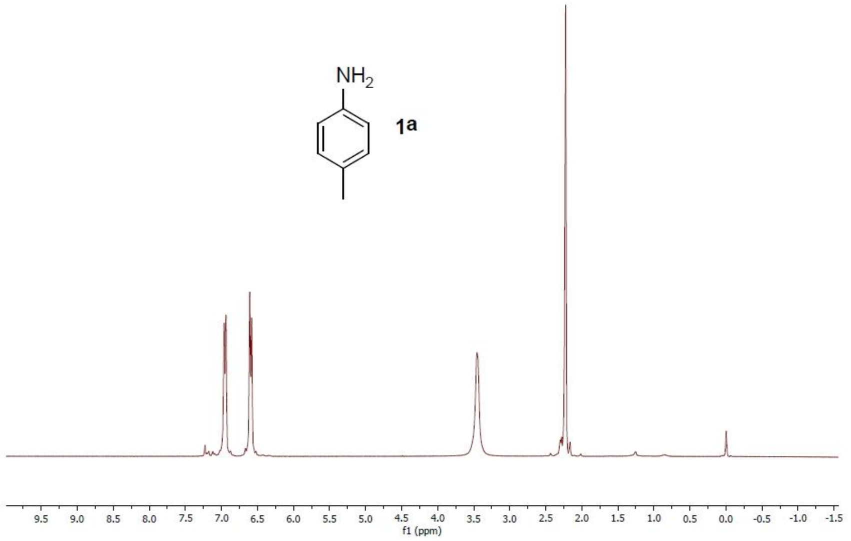

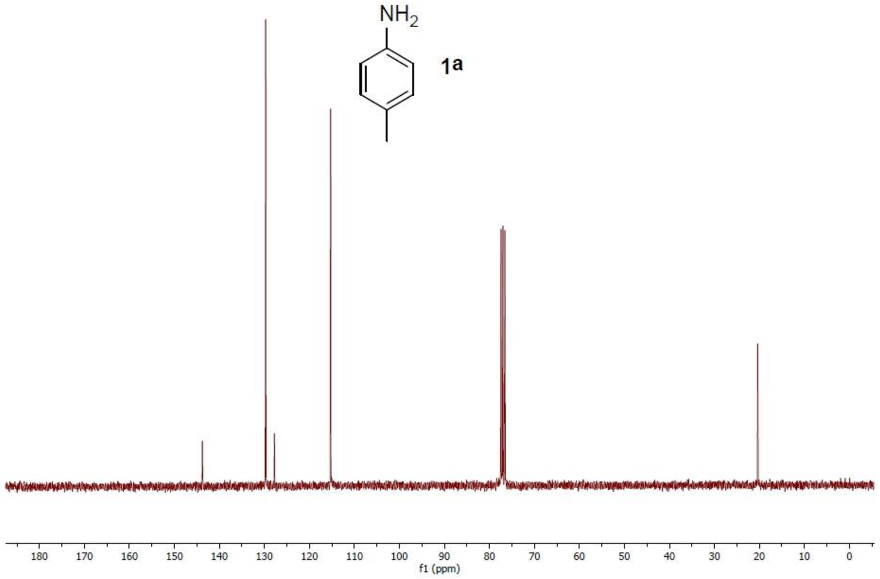

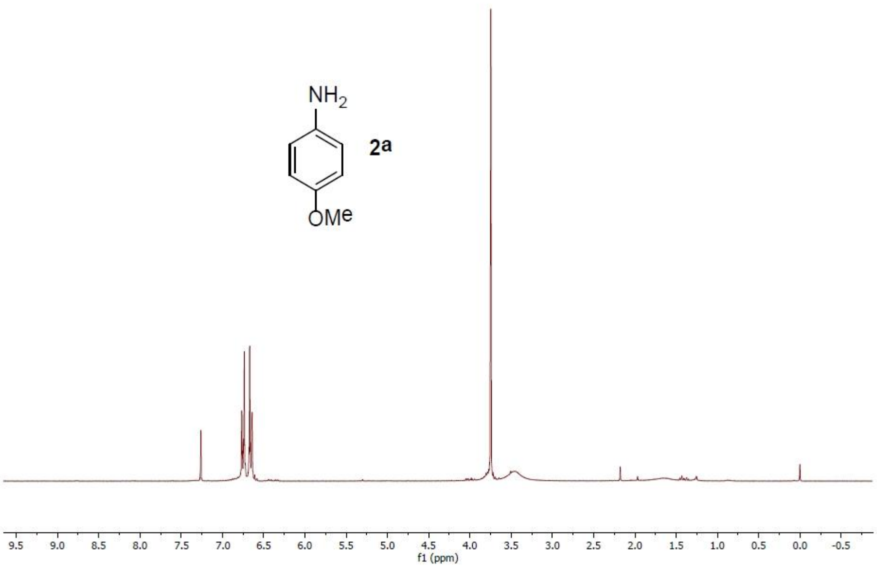
NH2
2a
OMe
9.5 9.0 8.5 8.0 7.5 7.0 6.5 6.0 5.5 5.0 4.5 4.0 3.5 3.0 2.5 2.0 1.5 1.0 0.5 0.0 -0.5
f1 (ppm)

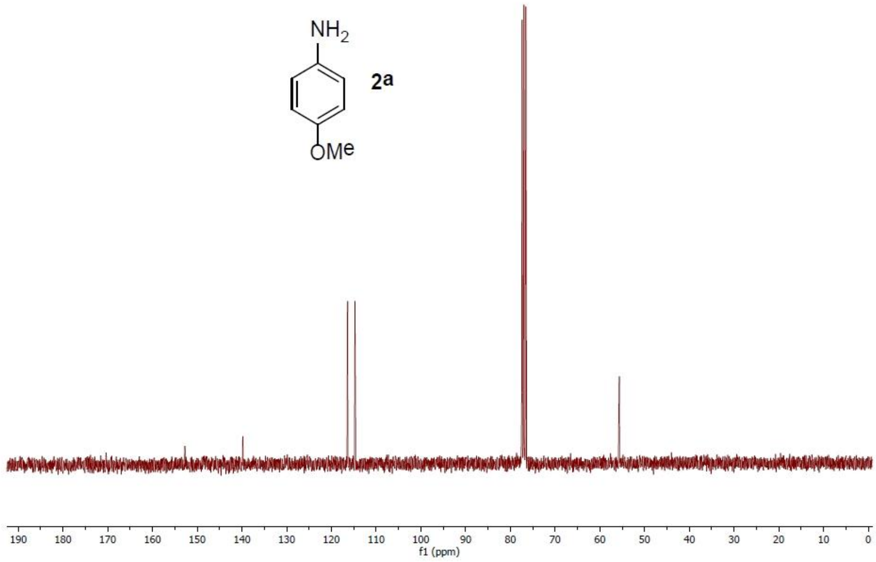
NH2
2a
OMe
190 180 170 160 150 140 130 120 110 100 90 80 70 60 50 40 30 20 10 0
f1 (ppm)

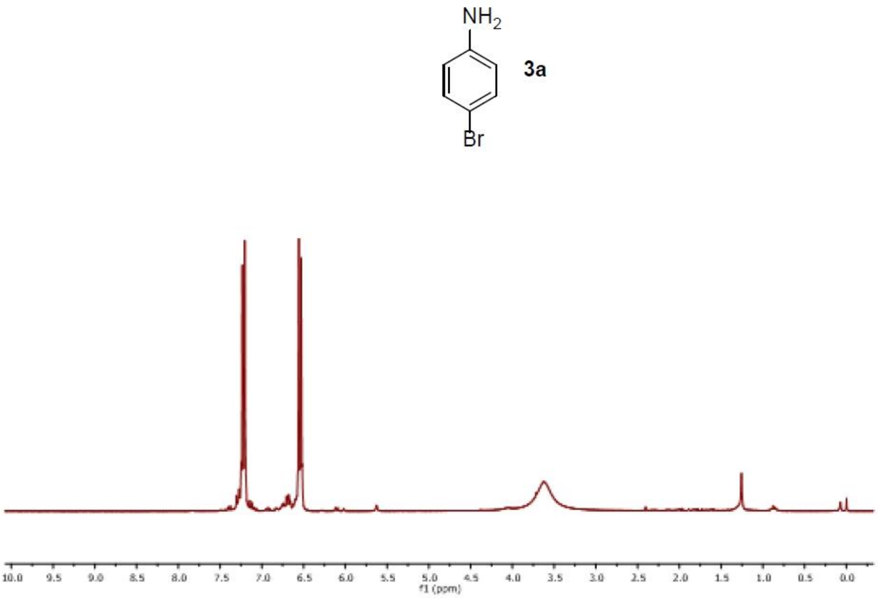
NH2
3a
Br
10.0 9.5 9.0 8.5 8.0 7.5 7.0 6.5 6.0 5.5 5.0 4.5 4.0 3.5 3.0 2.5 2.0 1.5 1.0 0.5 0.0
f1 (ppm)

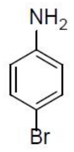
NH2
Br

3a

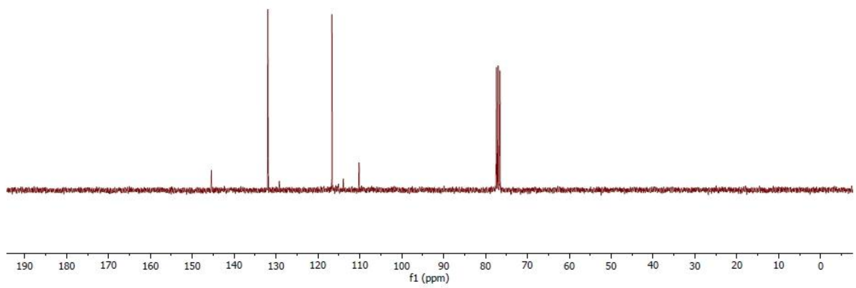
190 180 170 160 150 140 130 120 110 100 90 80 70 60 50 40 30 20 10 0
f1 (ppm)

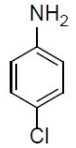
NH2
Cl

4a

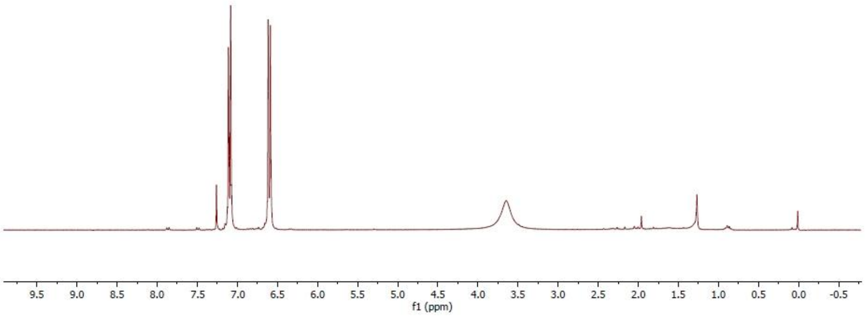
9.5 9.0 8.5 8.0 7.5 7.0 6.5 6.0 5.5 5.0 4.5 4.0 3.5 3.0 2.5 2.0 1.5 1.0 0.5 0.0 -0.5
f1 (ppm)

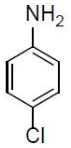
NH2
Cl

4a

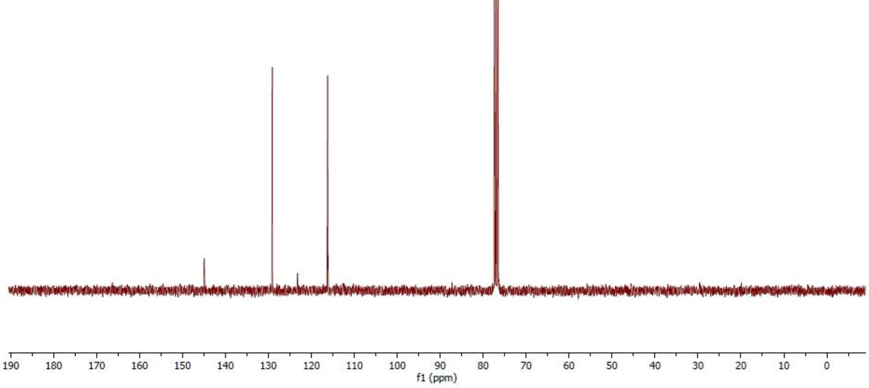
190 180 170 160 150 140 130 120 110 100 90 80 70 60 50 40 30 20 10 0
f1 (ppm)

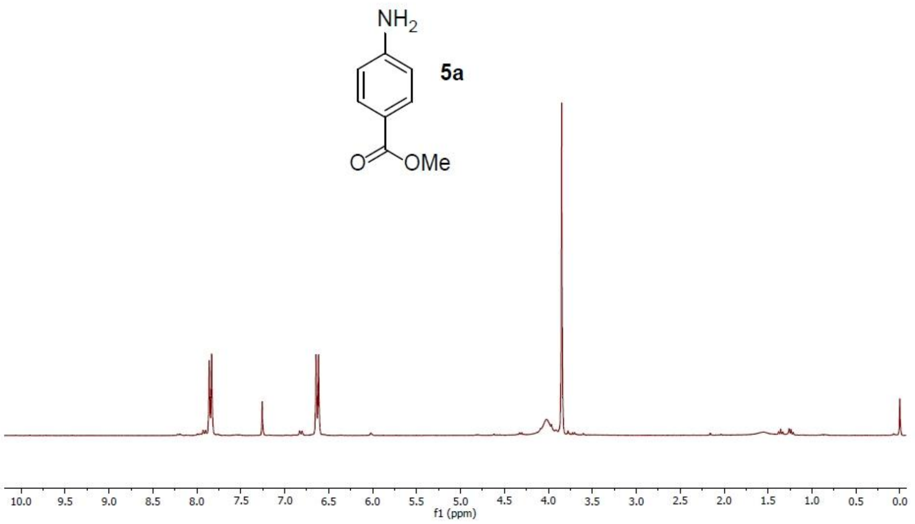
NH2
5a
OMe
10.0 9.5 9.0 8.5 8.0 7.5 7.0 6.5 6.0 5.5 5.0 4.5 4.0 3.5 3.0 2.5 2.0 1.5 1.0 0.5 0.0
f1 (ppm)

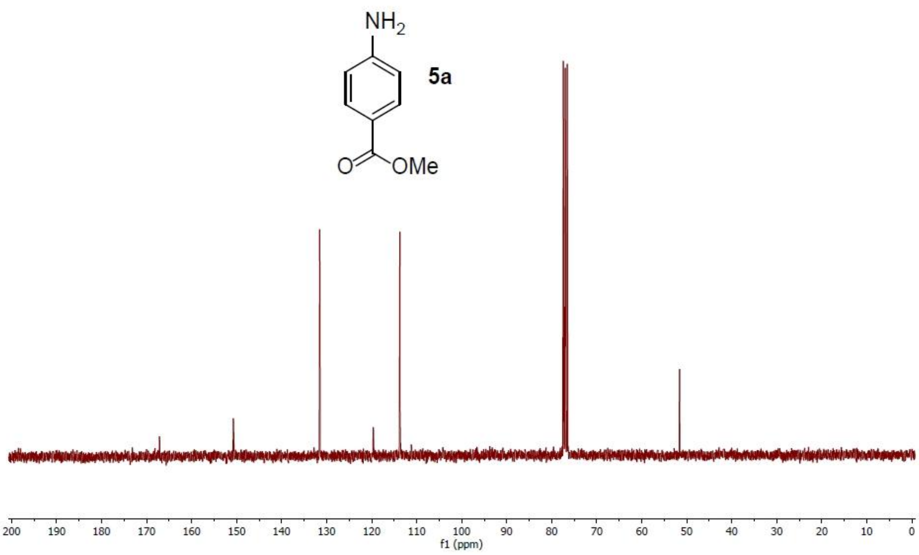
NH2
5a
OMe
200 190 180 170 160 150 140 130 120 110 100 90 80 70 60 50 40 30 20 10 0
f1 (ppm)

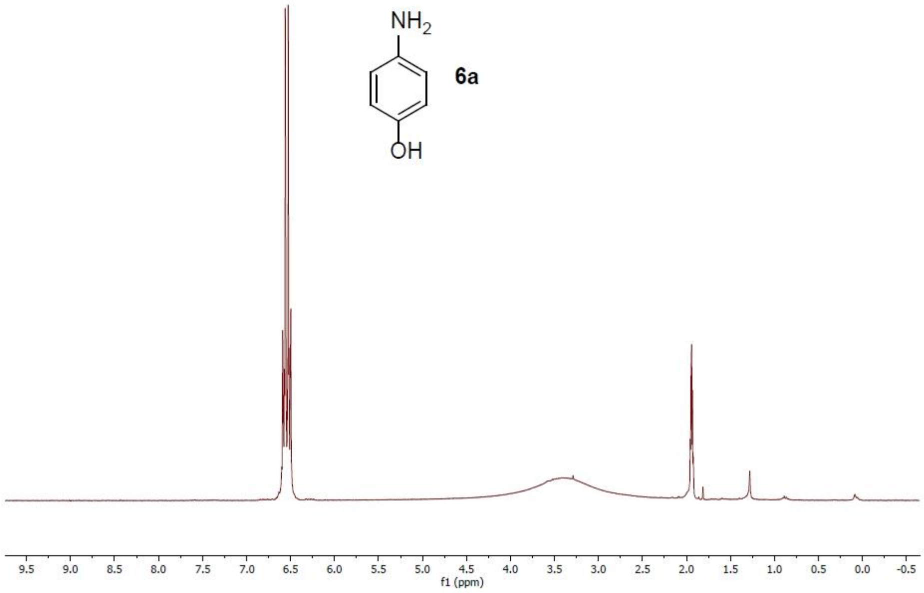
NH2
6a
OH
9.5 9.0 8.5 8.0 7.5 7.0 6.5 6.0 5.5 5.0 4.5 4.0 3.5 3.0 2.5 2.0 1.5 1.0 0.5 0.0 -0.5
f1 (ppm)

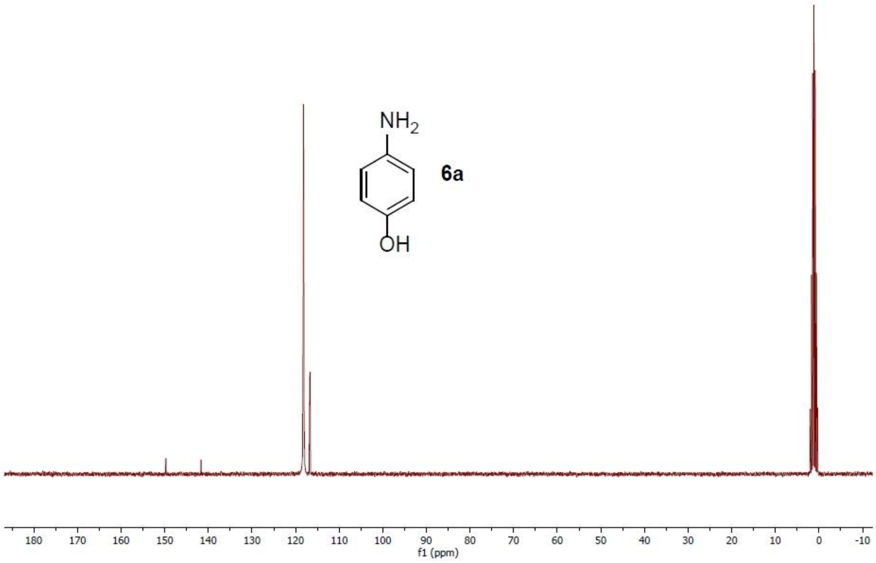
NH_2
OH
6a
180 170 160 150 140 130 120 110 100 90 80 70 60 50 40 30 20 10 0 -10
f1 (ppm)

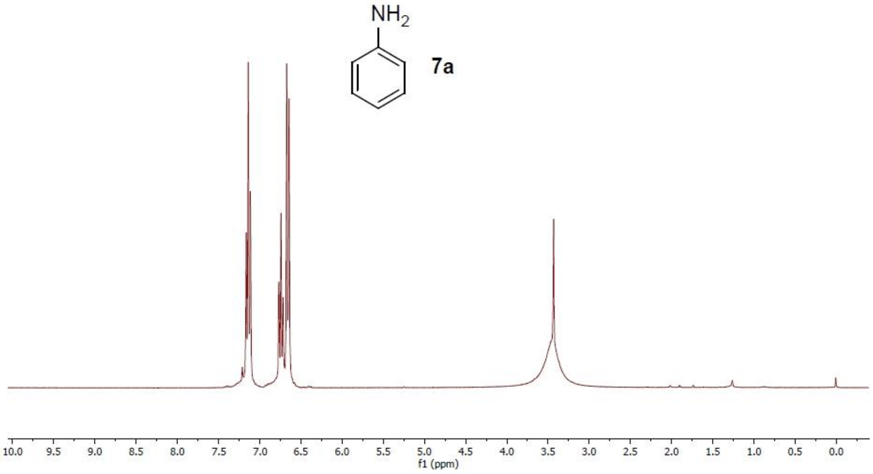
NH_2
7a
10.0 9.5 9.0 8.5 8.0 7.5 7.0 6.5 6.0 5.5 5.0 4.5 4.0 3.5 3.0 2.5 2.0 1.5 1.0 0.5 0.0
f1 (ppm)

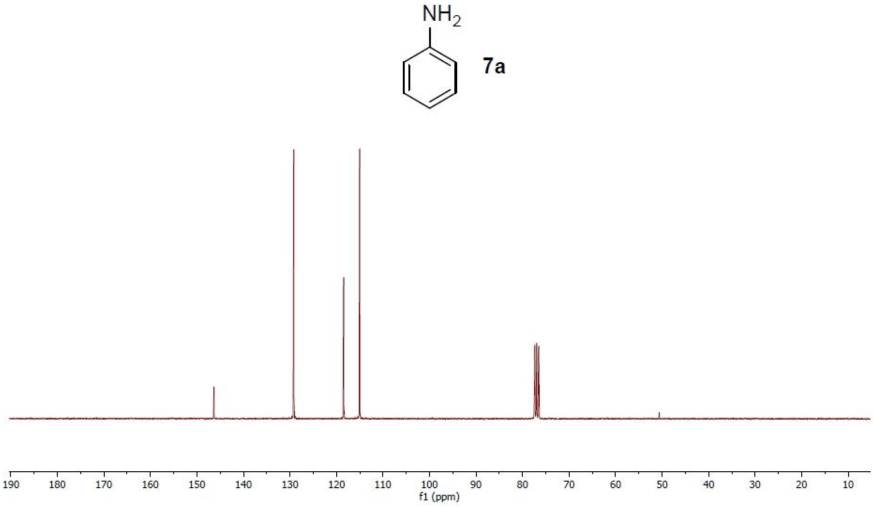
NH_2
7a
190 180 170 160 150 140 130 120 110 100 90 80 70 60 50 40 30 20 10
f1 (ppm)

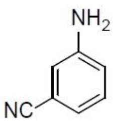

8a

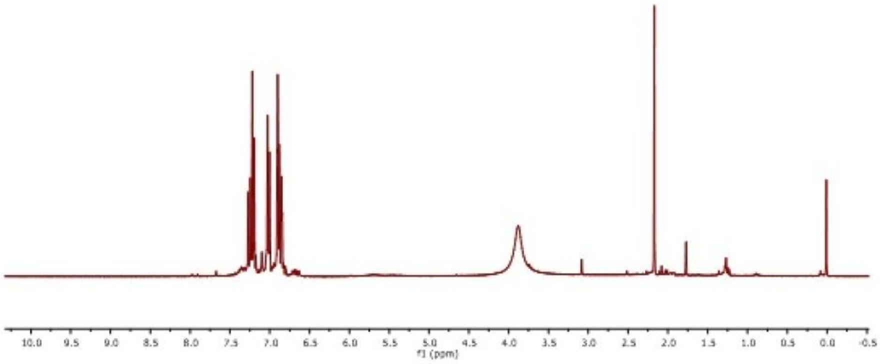

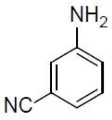

8a

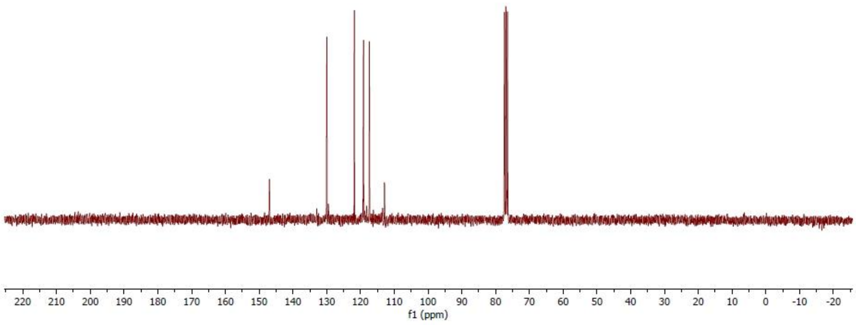

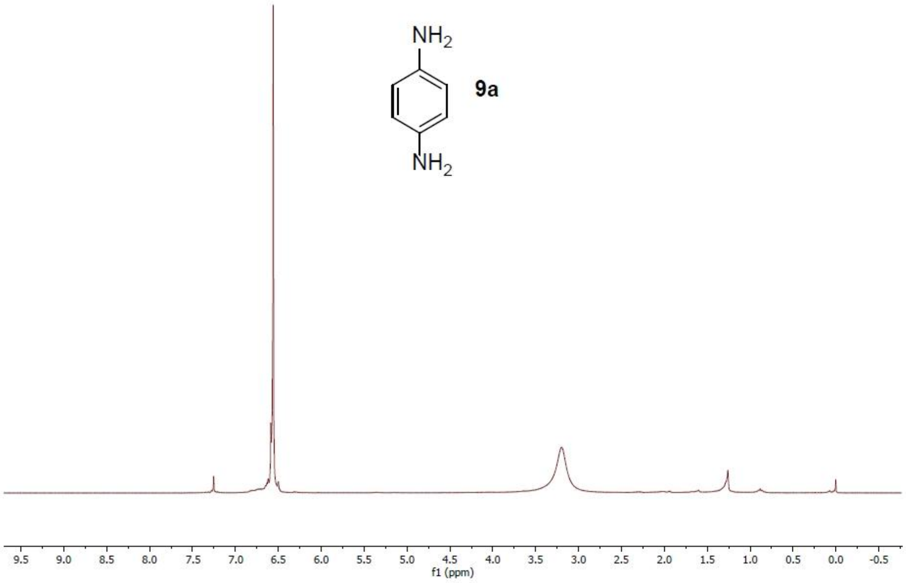

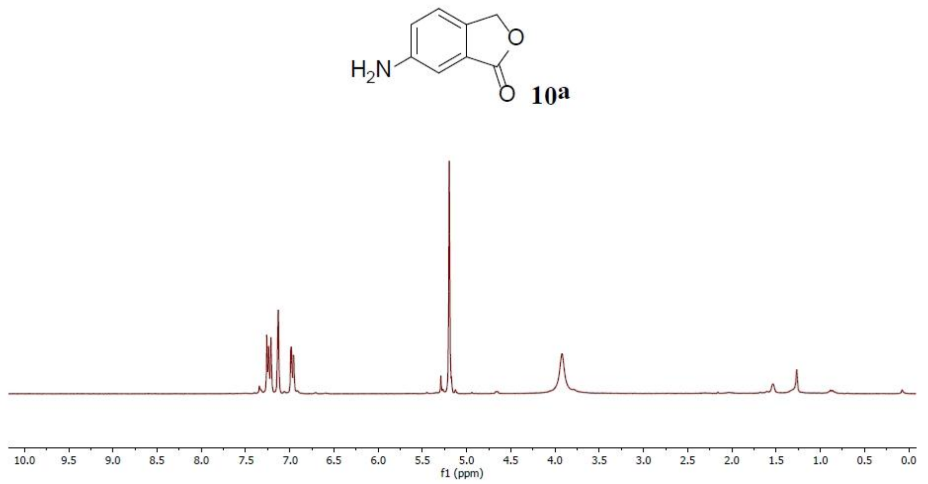
H2N
O
O
10a
f1 (ppm)

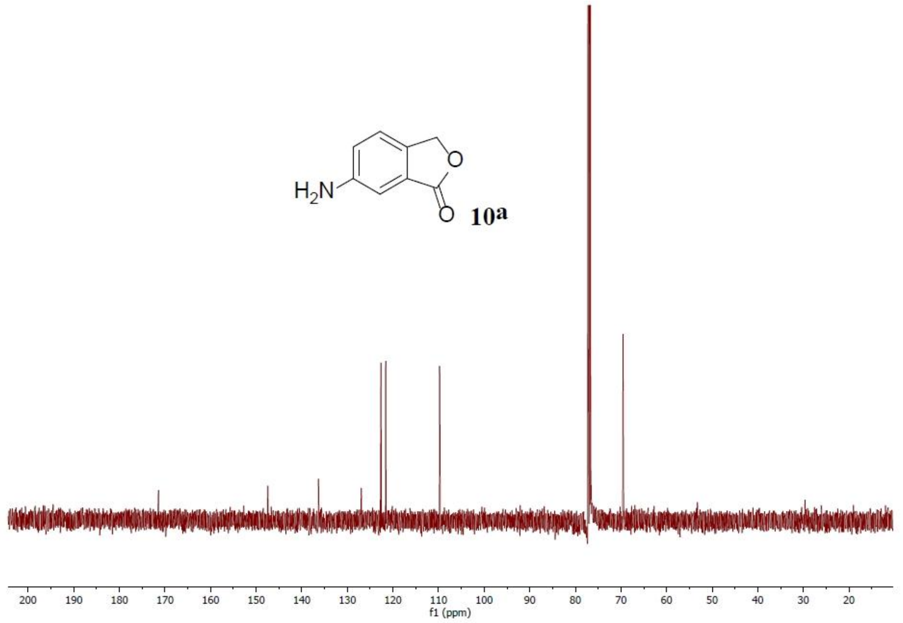
H2N
O
O
10a
f1 (ppm)

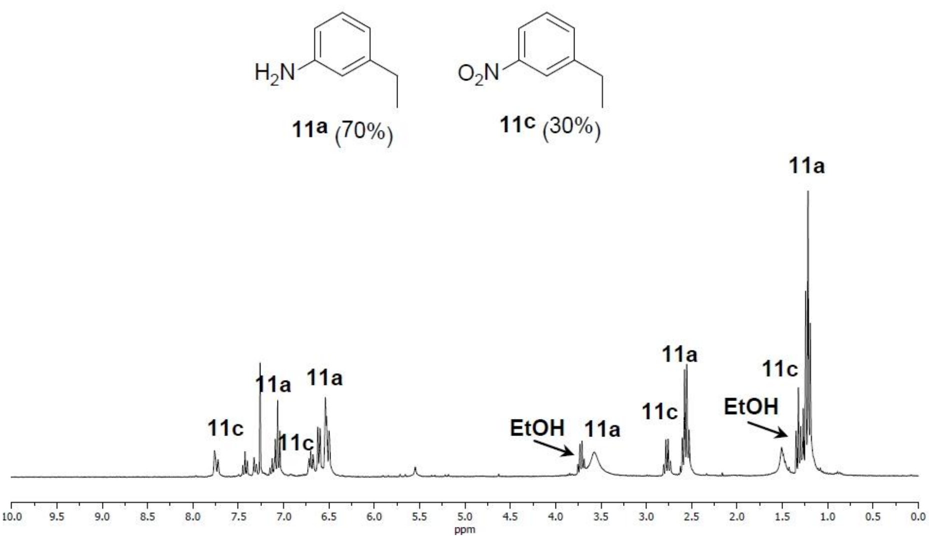
H2N
11a (70%)
O2N
11c (30%)
11a
11c
11a
11c
11a
EtOH
11a
11c
11a
11c
EtOH
ppm

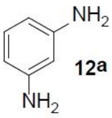
NH2
NH2

12a

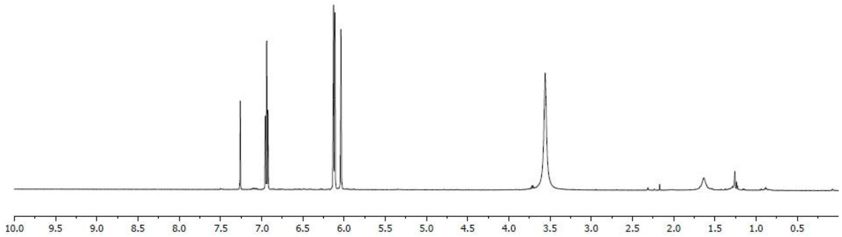
10.0 9.5 9.0 8.5 8.0 7.5 7.0 6.5 6.0 5.5 5.0 4.5 4.0 3.5 3.0 2.5 2.0 1.5 1.0 0.5

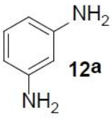
NH2
NH2

12a

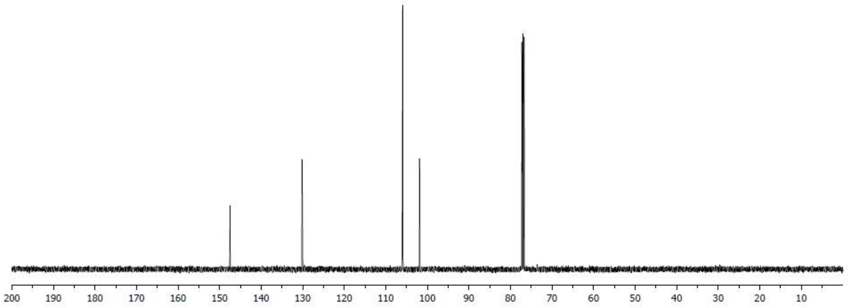
200 190 180 170 160 150 140 130 120 110 100 90 80 70 60 50 40 30 20 10

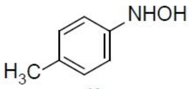
NHOH
H3C

1b

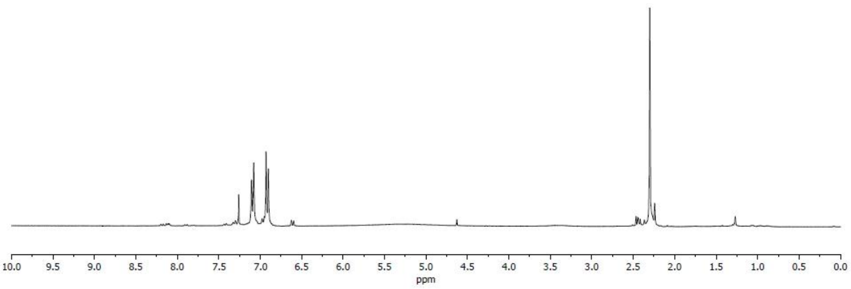
10.0 9.5 9.0 8.5 8.0 7.5 7.0 6.5 6.0 5.5 5.0 4.5 4.0 3.5 3.0 2.5 2.0 1.5 1.0 0.5 0.0
ppm

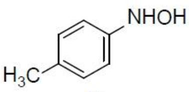

1b

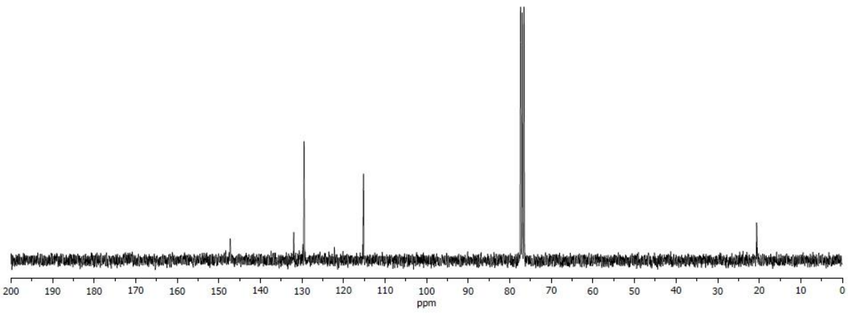

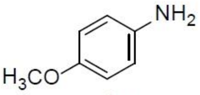

2a (15%)

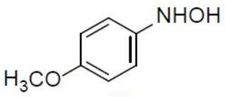

2b (85%)

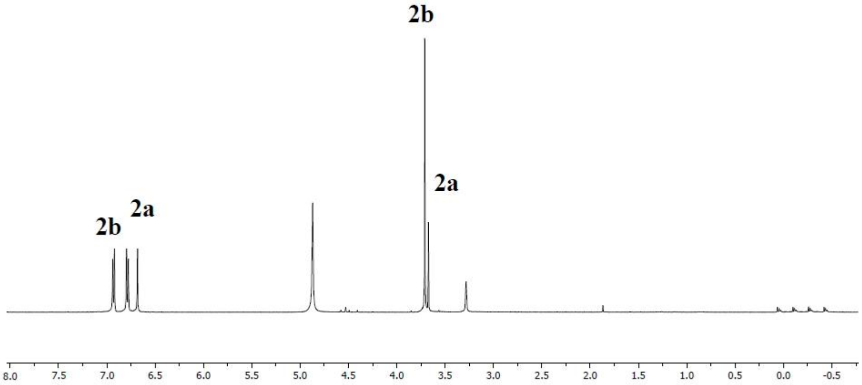

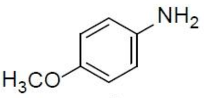

2a (15%)

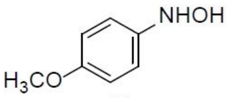

2b (85%)

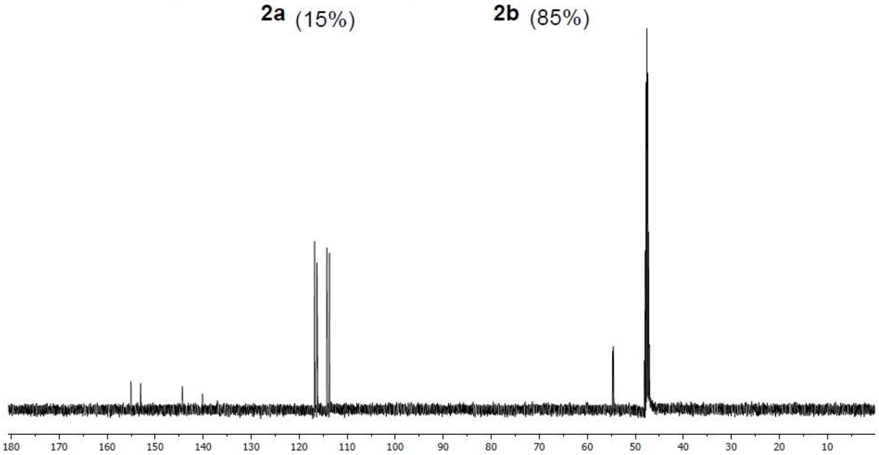

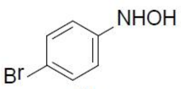
NHOH
Br

3b

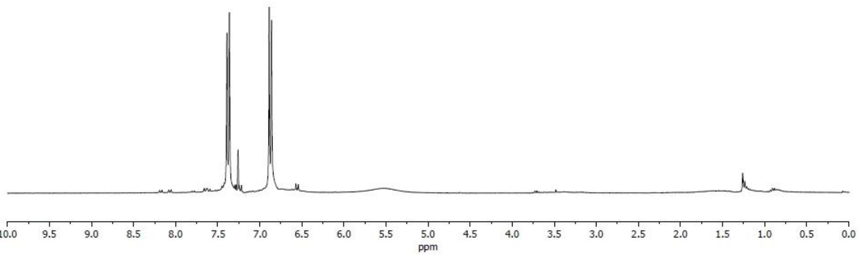
10.0 9.5 9.0 8.5 8.0 7.5 7.0 6.5 6.0 5.5 5.0 4.5 4.0 3.5 3.0 2.5 2.0 1.5 1.0 0.5 0.0
ppm

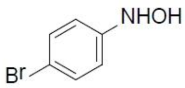
NHOH
Br

3b

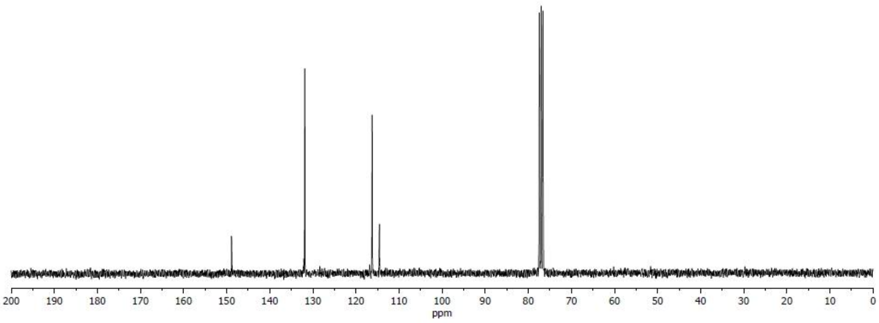
200 190 180 170 160 150 140 130 120 110 100 90 80 70 60 50 40 30 20 10 0
ppm

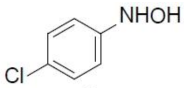
NHOH
Cl

4b

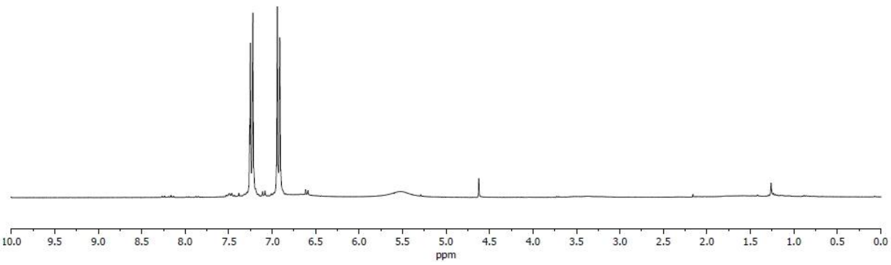
10.0 9.5 9.0 8.5 8.0 7.5 7.0 6.5 6.0 5.5 5.0 4.5 4.0 3.5 3.0 2.5 2.0 1.5 1.0 0.5 0.0
ppm

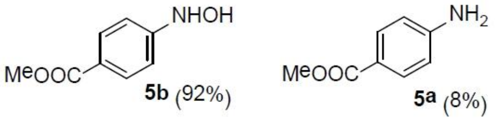
NHOH
MeOOC
5b (92%)
NH_2
MeOOC
5a (8%)

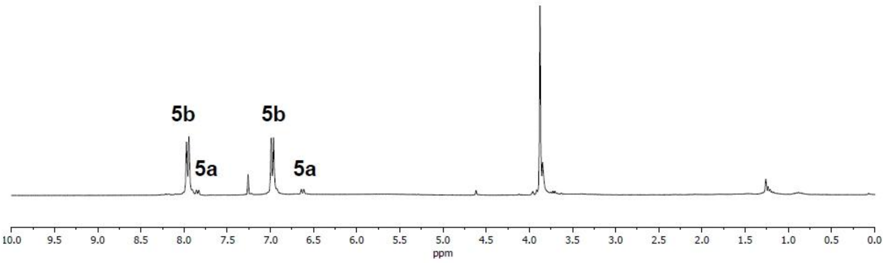
5b
5a
5b
5a
ppm

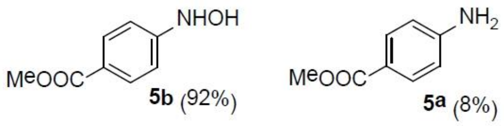
NHOH
MeOOC
5b (92%)
NH_2
MeOOC
5a (8%)

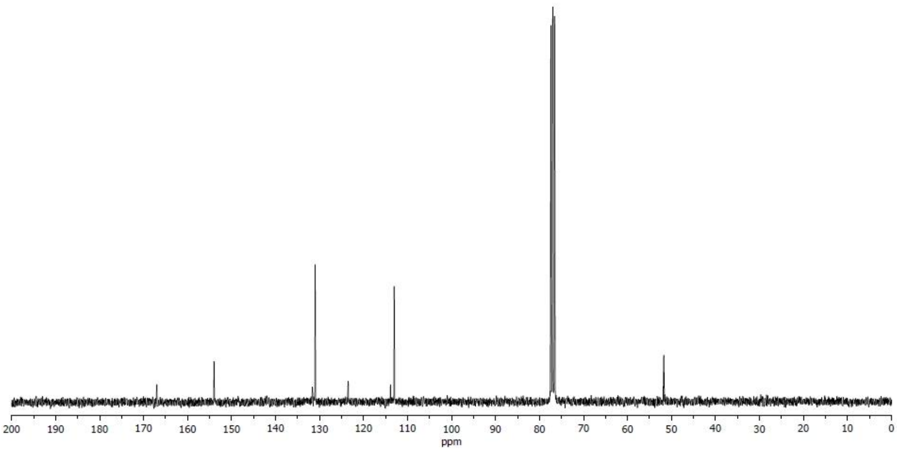
ppm

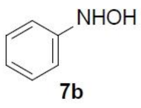
NHOH
7b

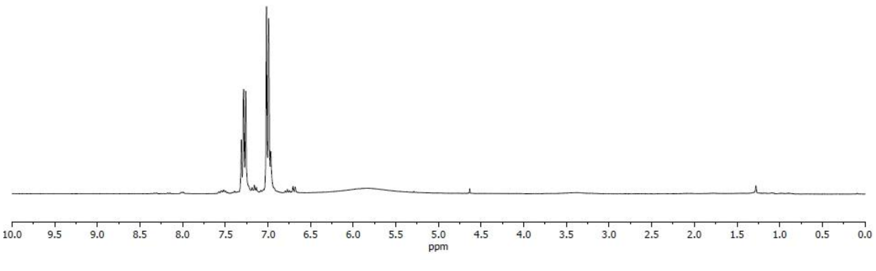
ppm

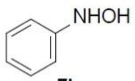

7b

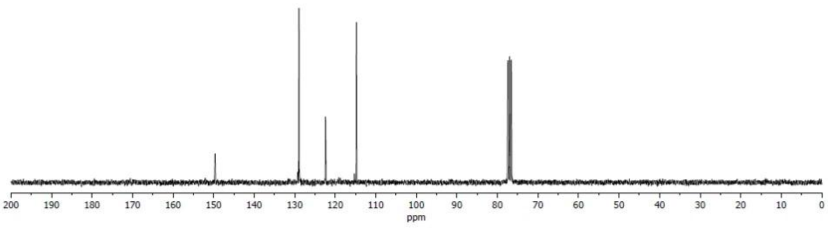

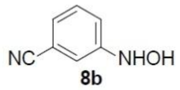

8b

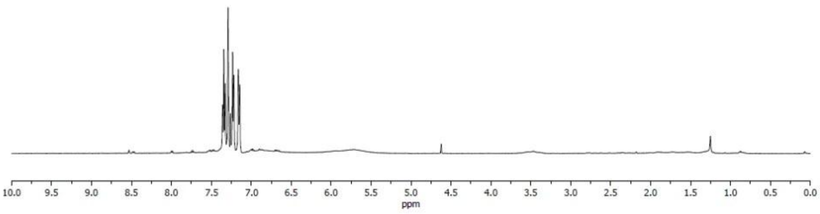

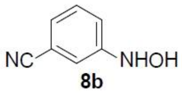

8b

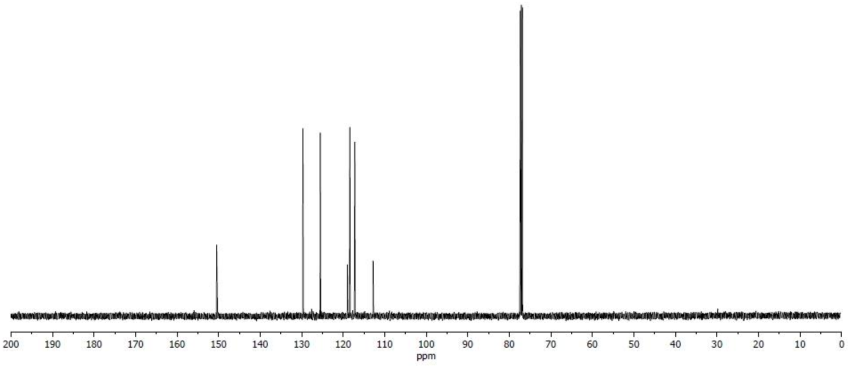

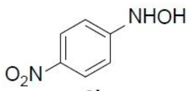

9b

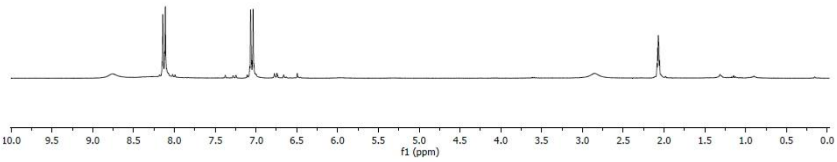

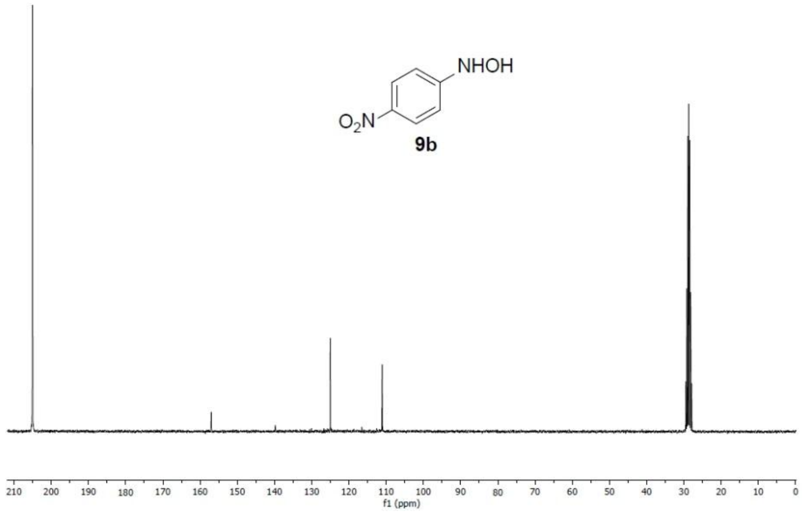

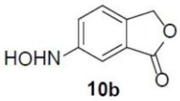

10b

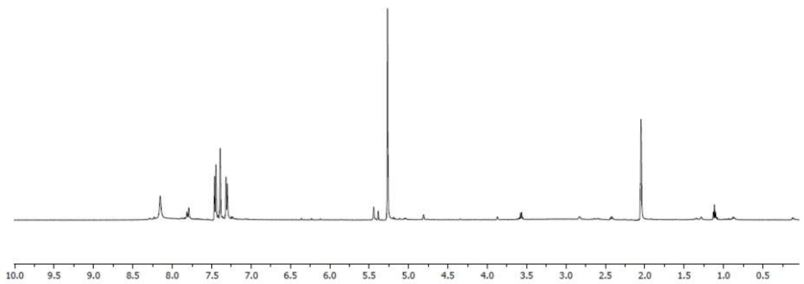

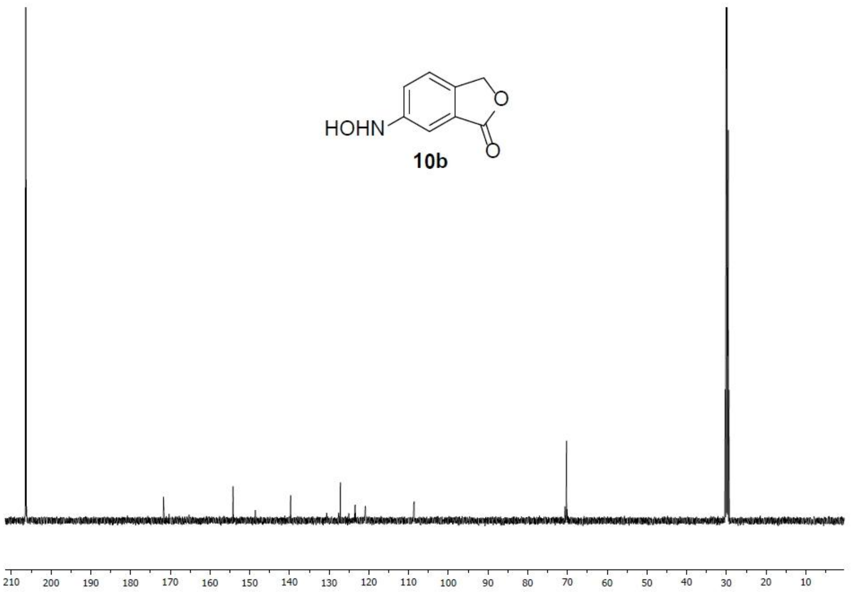
HOHN
O
O
10b

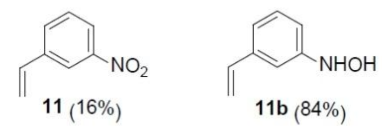
NO2
NHOH
11 (16%)
11b (84%)

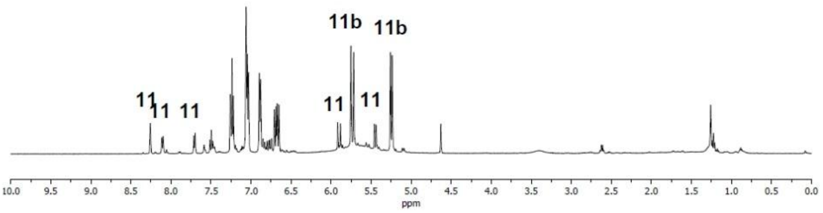
11b
11b
11
11
11
11
11
ppm

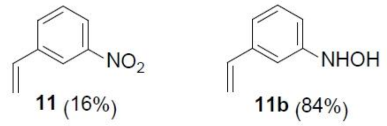
NO2
NHOH
11 (16%)
11b (84%)

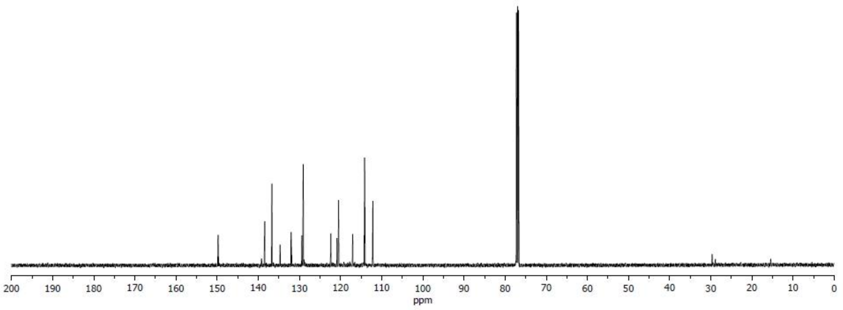
ppm

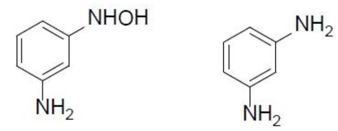

12b (70%) **12a** (30%)

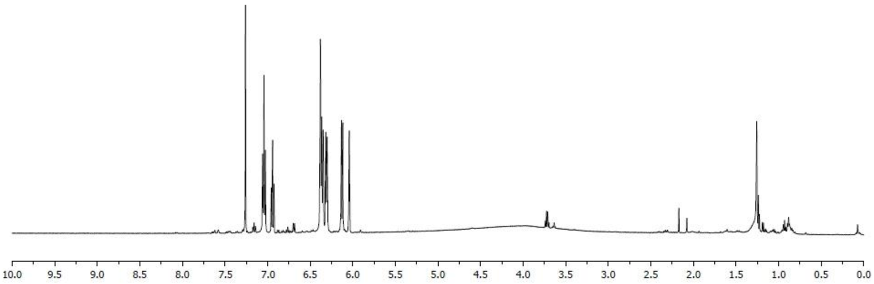

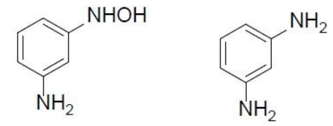

12b (70%) **12a** (30%)

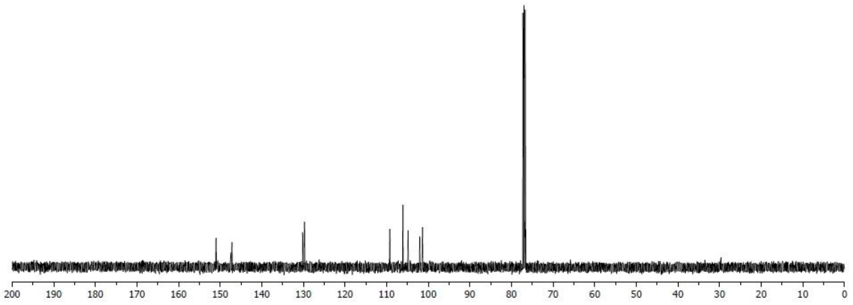

References

1. Lowry, T.H.; Richardson, K.S. *Mechanism and Theory in Organic Chemistry*, 3rd ed.; Harper and Row Inc.: New York, NY, USA, 1987.
2. Sharma, U.; Verma, P.K.; Kumar, N.; Kumar, V.; Bala, M.; Singh, B. Phosphane-free green protocol for selective nitro reduction with an Iron-based catalyst. *Chem. Eur. J.* **2011**, *17*, 5903–5907.
3. Sharma, U.; Kumar, N.; Verma, P.K.; Kumar, V.; Singh, B. Zinc phthalocyanine with PEG-400 as a recyclable catalytic system for selective reduction of aromatic nitro compounds. *Green Chem.* **2012**, *14*, 2289–2293.
4. Dey, R.; Mukherjee, N.; Ahammed, S.; Ranu, B.C. Highly selective reduction of nitroarenes by iron(0) nanoparticles in water. *Chem. Commun.* **2012**, *48*, 7982–7984.
5. Rahaim, R.J.; Maleczka, R.E. Pd-catalyzed silicon hydride reductions of aromatic and aliphatic nitro groups. *Org. Lett.* **2005**, *7*, 5087–5090.
6. Chapman, N.; Conway, B.; O'Grady, F.; Wall, M.D. A convenient method to aniline compounds using microwave-assisted transfer hydrogenation. *Synlett* **2006**, *7*, 1043–1046.
7. Takenaka, Y.; Kiyosu, T.; Choi, J.-C.; Sakakura, T.; Yasuda, H. Selective synthesis of *N*-aryl hydroxylamines by the hydrogenation of nitroaromatics using supported platinum catalysts. *Green Chem.* **2009**, *11*, 1385–1390.
8. Boymans, E.H.; Witte, P.T.; Vogt, D. A study on the selective hydrogenation of nitroaromatics to *N*-arylhydroxylamines using a supported Pt nanoparticle catalyst. *Catal. Sci. Technol*. **2015**, *5*, 176–183.

© 2016 by the authors; licensee MDPI, Basel, Switzerland. This article is an open access article distributed under the terms and conditions of the Creative Commons by Attribution (CC-BY) license (http://creativecommons.org/licenses/by/4.0/).
